# Supplementary material for: miR-22 gene therapy treats HCC by promoting anti-tumor immunity and enhancing metabolism
Source: Mol Ther. 2023 May 4;31(6):1829–45. doi: 10.1016/j.ymthe.2023.04.019 (PMC10277895; doi:10.1016/j.ymthe.2023.04.019)
Supplement: Document S1. Figures S1–S7 and Tables S1–S3 [file mmc1.pdf]

YMTHE, Volume *31*

Supplemental Information

miR-22 gene therapy treats HCC by promoting anti-tumor immunity and enhancing metabolism

Ying Hu, Tahereh Setayesh, Farzam Vaziri, Xuesong Wu, Samuel T. Hwang, Xin Chen, and Yu-Jui Yvonne Wan

Table S1. Antibodies used for IHC, Western blot, ChIP, or flow cytometry.

Name	Citation	Supplier	Cat no.	Clone no.
IL6Rα	28	Santa Cruz Biotechnology	sc-660	
Phospho-Stat3 (Tyr705)	1236	Cell Signaling Technology	#9131	
Stat3 (124H6) Mouse mAb	1338	Cell Signaling Technology	#9139	124H6
IL17A	20	Invitrogen eBioscience	14-7175-81	eBio17CK15A5
HIF1α	862	Novus Biologicals	NB100-105	H1alpha67
β-ACTIN	3568	MilliporeSigma	A1978	
Ki-67	264	NeoMarkers	RB-1510-P	
CD45-PE/Cy7	144	Biolegend	103114	30-F11
CD3-BuV395	30	BD Biosciences	563565	145-2C11
CD4-BV605	17	BD Biosciences	563151	RM4-5
CD8-Alexa700	27	BD Biosciences	557959	53-6.7
CD44-BV711	9	BD Biosciences	563971	IM7
CD62L-Alexa647	8	Biolegend	104421	MEL-14
CD25-PE	290	Invitrogen eBioscience	12-0251-82	PC61.5
FoxP3-FITC	376	Invitrogen eBioscience	11-5773-82	FJK-16s
IL17A-PECF594	6	BD Biosciences	562542	TC11-18H10
CD107a-BV711	0	BD Biosciences	564348	1D4B
CD45-PE	125	Biolegend	103106	30-F11
CD3-PerCP Cy5.5	67	BD Biosciences	551163	145-2C11
CD8-FITC	32	Invitrogen	MA5-17597	CT-CD8a
CD4-APC	161	Biolegend	100412	GK1.5
InVivoMAb anti-mouse CD8α	80	Bio X Cell	BE0004-1	53-6.7
InVivoMAb rat IgG2a isotype control	367	Bio X Cell	BE0089	
IFNγ-PE	2	BD Biosciences	612769	XMG1.2
CD3-APC	132	BD Biosciences	553066	145-2C11
RORγ	129	Invitrogen eBioscience	12-6988-82	AFKJS-9
Normal Mouse IgG	329	MilliporeSigma	13-371	
Normal Rabbit IgG	641	MilliporeSigma	12-370	
RNA Polymerase II	195	MilliporeSigma	05-623	CTD4H8

Table S2. Primer sequences.

Primers	Sequence	
Primers used for qRT-PCR		
Afp	F 5’-CAGTGCGTGACGGAGAAGAA -3’	R 5’-AAACACCCATCGCCAGAGTT-3’
Cd133	F 5’-TGATTCCAAGGAGATTGCCCT-3’	R 5’-GCAGCAACGGCACATACAAA-3’
Ccna2	F 5’-ACAGAGCTGGCCTGAGTCAT-3’	R 5’-TTGACTGTTGGGCATGTTGT-3’
Gpc3	F 5’-AATCAACTGCGCTTCCTTGC-3’	R 5’-AGGTGGTGATCTCGTTGTCC-3’
Alb	F 5’-TACAGCGGAGCAACTGAAGA-3’	R 5’-TTGCAGCACAGAGACAAGAA-3’
Cyp3a11	F 5’-TCACAGACCCAGAGACGATTAAGA-3’	R 5’-CCCGCCGGTTTGTGAAG-3’
Cd3e	F 5’- TCTCGGAAGTCGAGGACAGT3’	R 5’-ATCAGCAAGCCCAGAGTGAT-3’
Cd4	F 5’- ACACACCTGTGCAAGAAGCA-3’	R 5’-GCTCTTGTTGGTTGGGAATC-3’
Cd8a	F 5’-CTCACCTGTGCACCCTACC-3’	R 5’-ATCCGGTCCCCTTCACTG-3’
Il17a	F 5’-TTTAACTCCCTTGGCGCAAAA-3’	R 5’-CTTTCCCTCCGCATTGACAC-3’
Il17f	F 5’-CTGGAGGATAACACTGTGAGAGT-3’	R 5’-TGCTGAATGGCGACGGAGTTC-3’
Rorc	F 5’-TCCCGAGATGCTGTCAAGTT-3’	R 5’-ACTTGTTCCTGTTGCTGCTG-3’
Ccl20	F 5’-ACTGTTGCCTCTCGTACATACA-3’	R 5’-ACCCACAATAGCTCTGGAAGG-3’
Ccr6	F 5’-GTCACTGTCATGCTTACTTGAATG-3’	R 5’-CTTAGGACTGGAGCCTGGATA-3’
Il6	F 5’-GTTGCCTTCTTGGGACTGATG-3’	R 5’-GGGAGTGGTATCCTCTGTGAAGTCT-3’
Il6ra	F 5’-ACAGTGTGGGAAGCAAGTCC-3’	R 5’-TCGGTATCGAAGCTGGAACT-3’
Il23a	F 5’-TGAAGATGTCAGAGTCAAGCAG-3’	R 5’-ACAAGGACTCAAGGACAACAG-3’
Il23r	F 5’-AAGGCTTTTCGGAACCTCAT-3’	R 5’-TTCCAGGTGCATGTCATGTT-3’
Il22	F 5’-TTGAGGTGTCCAACTTCCAGCA -3’	R 5’-AGCCGGACGTCTGTGTTGTTA-3’
S100a8	F 5’-TGTCCTCAGTTTGTGCAGAATATAAA-3’	R 5’-TCACCATCGCAAGGAACTCC-3’
S100A9	F 5’-GGTGGAAGCACAGTTGGCA-3’	R 5’-GTGTCCAGGTCCTCCATGATG-3’
Hif1a	F 5’-TCATCCATGTGACCATGAGG-3’	R 5’-AAAAAGCTCCGCTGTGTGTT-3’
Tgfb1	F 5’- GCCTGAGTGGCTGTCTTTTGACG-3’	R 5’-ACTTCCAACCCAGGTCCTTC-3’
Foxp3	F 5’-TCCTTCCCAGAGTTCTTCCA-3’	R 5’-CGAACATGCGAGTAAACCAA -3’
Il2ra	F 5’-AACGGCACCATCCTAAACTG-3’	R 5’-CTGTGTTGGCTTCTGCATGT-3’
Il2	F 5’-AGGAACCTGAAACTCCCCAG-3’	R 5’- AAATCCAGAACATGCCGCAG-3’
Nt5e	F 5’-AGGTTGTGGGGATTGTTGGA-3’	R 5’-CCCCAGGGCGATGATCTTAT-3’
Lag3	F 5’-CCTCGATGATTGCTAGTCCCT-3’	R 5’-GTAGACAGGCACTCGGTTCTG-3’
Nrp1	F 5’-AATGTTCTGTCGCTATGACCGGCT-3’	R 5’-TTCTGCCCACAATAACGCCCAATG-3’
Ctla4	F 5’-CATGTACCCACCGCCATACT-3’	R 5’-CCAAGCTAACTGCGACAAGG-3’
Il10	F 5’-GGAGCAGGTGAAGAGTGATTTTAATA-3’	R 5’-TGCAGTTGATGAAGATGTCAAATTC-3’
Il12a	F 5’-TGATGATGACCCTGTGCCTT-3’	R 5’-CGCAGAGTCTCGCCATTATG-3’
Il35b	F 5’-GATCCACGTCCTTCATTGCC-3’	R 5’-TGATTCGCTCAGCCACAAAG-3’
Runx1	F 5’-AGCGAGATTCAACGACCTCA-3’	R 5’-GCCGTCCACTGTGATTTTGA-3’
Itga4	F 5’-GCCTGGAGGAGAGGGATAAC-3’	R 5’-CAGAAGGCATGACGTAGCAA-3’
Itgb7	F 5’-CTACGACTCTGGGCTCTTGG-3’	R 5’-ACAGGTCAGCCTCAGAGCAT-3’
CCr9	F 5’-CTTGCCACTCTTCCCTTCTG-3’	R 5’-GCCTTCATGGCCTGTACAAT-3’
Ccl25	F 5’-CCAAGGTGCCTTTGAAGACT-3’	R 5’-TCCTCCAGCTGGTGGTTACT-3’
Madcam1	F 5’-GCATGGTGACCTGGCAGTGAAG-3’	R 5’-GGCAGCAGTATCCTCTCTGTAC-3’

Ifih1	F 5'-GGAAACAGCGGGAATGAGTC-3'	R 5'-AGCAGGCAGAAGACACTCAT-3'
Dhx58	F 5'-GTAGACAGAGGCAAGGTGGT-3'	R 5'-TACAGATGAGCAGGTCGTGG-3'
Rarb	F 5'-GCACTGACGCCATAGTGGTA-3'	R 5'-CACCATCTCCACTTCCTCCT-3'
Cyp26a1	F 5'-GCACAAGCAGCGAAAGAAGGTGAT-3'	R 5'-ACTGCTCCAGACAACTGCTGACTT-3'
Cyp26b1	F 5'-CGGAGAGACTGGTCACTGGT-3'	R 5'-CGCCCCAGTAAGTGTGTCTT-3'
Gapdh	F 5'-TGTGTCCGTCGTGGATCTGA-3'	R 5'-CCTGCTTCACCACCTTCTTGA-3'
18s	F 5'-CCGAAGCGTTTTACTTTGAAAAAA-3'	R 5'-TTCATTATTCCTAGCTGCGGTATC-3'
miR-22	F 5'-CGCGAAGCTGCCAGTTGAAG-3'	R 5'-GTGCAGGGTCCGAGGT-3'
U6	F 5'-CTCGCTTCGGCAGCACA-3'	R 5'-AACGCTTCACGAATTTGCGT-3'

Primers used for ChIP-qPCR

RORγt _P1 (-4149 to -4208) HIF1α- ChIP	F 5'-CACCTCCAGGTTGTTTGCCCC-3'
	R 5'-GAGTGTGCATGTCTGTGGAGG-3'
RORγt_P2 (-6988 to -7002) HIF1α- ChIP	F 5'-GCGAAGGGACAGCTGCCTGC-3'
	R 5'-CTCCAGCTGGTAAACAGCAG-3'
RORγt _P3 (non-HIF1 binding) HIF1α- ChIP	F 5'-CAATCCTCCGTGCTGACAGCA-3'
	R 5'-CTGTCTAAGGGCGAAGGTCA-3'
IL17A _P1 (-4739 to -4755) p-STAT3-ChIP	F 5'- CAGGTATTATTCTCAGGGCTTTGG -3'
	R 5'- TGGCAATGGTGTCTTTTCTTTG -3'
IL17A _P2 (-19 to -32) p-STAT3-ChIP	F 5'- CACCTCACACGAGGCACAAG-3'
	R 5'- ATGTTTGCGCGTCCTGATC-3'
IL-17A_P3 (-2913 to -2925) RORγt or HIF1α- ChIP	F 5'- GGCTACAACATAGTCATACAC-3'
	R 5'- GAAGGTATCAGATCCCATTAC-3'
IL-17A_P4 (-6128 to -6140) RORγt or HIF1α- ChIP	F 5'- CAGATGCATGCAGAACTGAC-3'
	R 5'- AGGCTCTGGAGAGCAGACA-3'
IL-17A_P5 (non- RORγt/P-STAT3) binding) RORγt or p-STAT3- ChIP	F 5'- AGTCTGGCCCCTACACACAC-3'
	R 5'- ATGGGGGACTTTTGGGATAG-3'

Table S3. Mouse HCC scoring.

	Features				
Score	Centrilobular vacuolar degeneration	Foci of proliferation	Mitotic rate	Scirrhous type foci of proliferation	Inflammatory cell infiltration
1	<10% of lobules	Small with vacuolated cells (<1 focus / 10X HPF[1])	<1 / 40X HPF[1]	1 or 2 / 10X HPF[1]	<1 / 20x HPF[1]
2	10~33% of lobules	Moderate-sized, multifocal, with small non-vacuolated cells (1 to 2 foci / 10X HPF[1])	1 to 3 / 40X HPF[1]	3 to 6 / 10X HPF[1]	1 to 2 / 20x HPF[1]
3	33~66% of lobules	Moderate-sized and coalescing or large, multifocal (2 to 3 foci / 10X HPF[1])	4 to 6 / 40X HPF[1]	7 to 10 / 10X HPF[1]	2 to 3 /20x HPF[1]
4	>66% of lobules	large and coalescing) >3 foci / 10X HPF[1]	>6 / 40X HPF[1]	>10 / 10X HPF[1]	>3 / 20x HPF[1]

[1] HPF: high-power field.

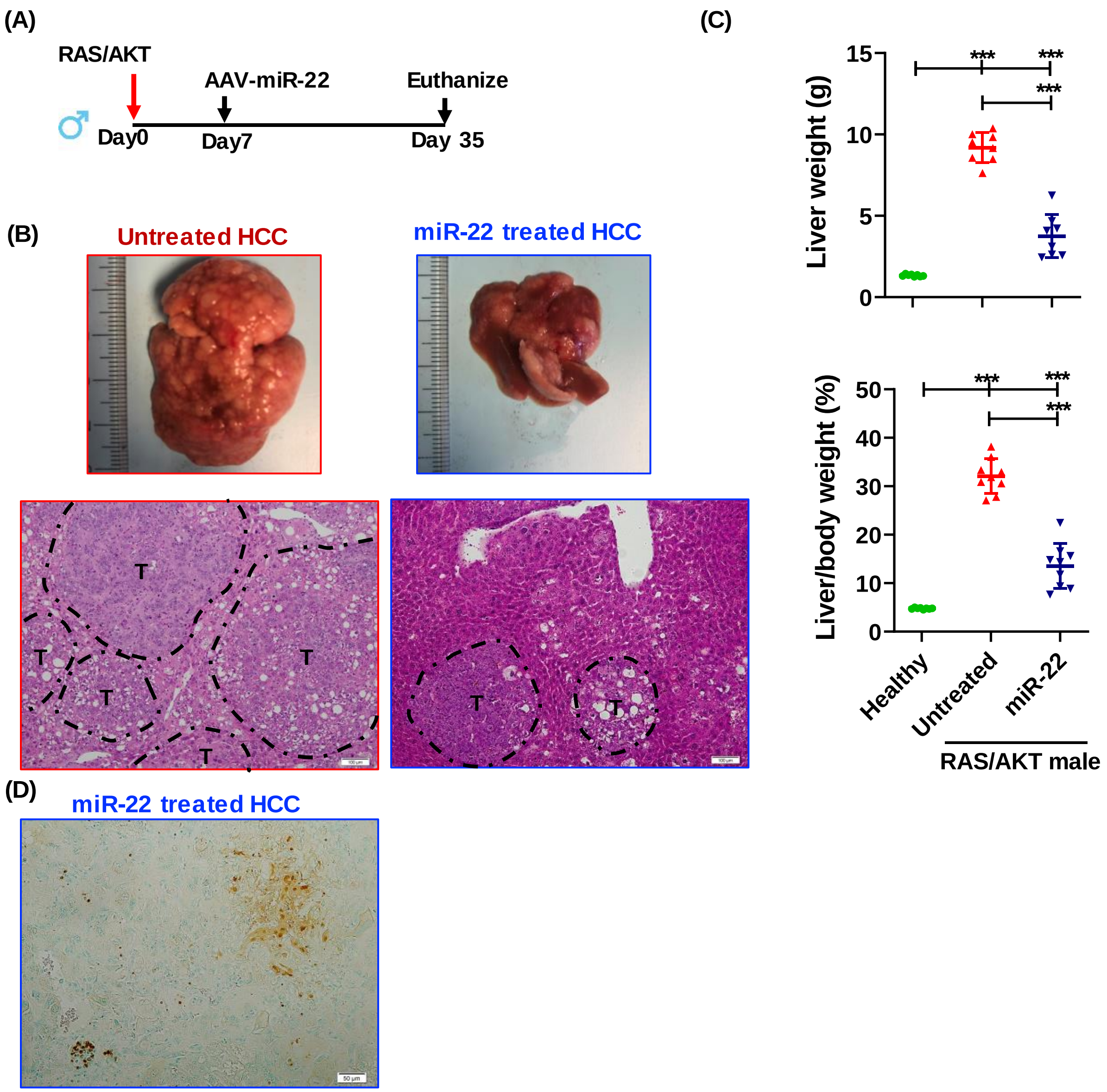

Figure S1. miR-22 treats HCC in male mice. **(A)** Study design for miR-22 treatment in RAS/AKT-induced HCC model, **(B)** Representative liver morphology and H&E-stained liver sections (10X), **(C)** Liver weight and L/B ratio for the studied groups. **(D)** Apoptosis detected by TUNEL staining was found in miR-22-treated HCC female mice as one example (20X).

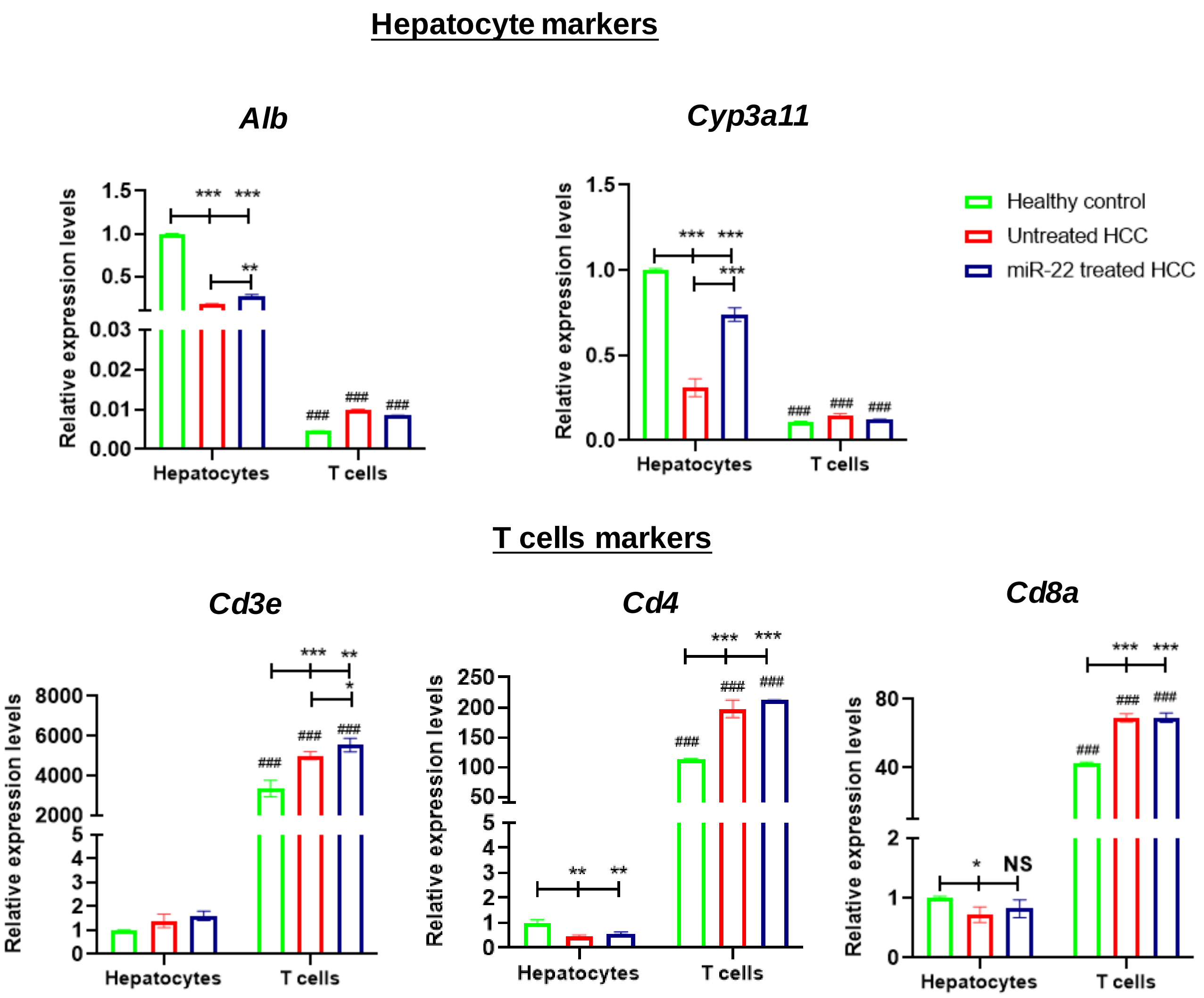

Figure S2. The expression levels of hepatocyte and T cell markers in isolated hepatocytes and T cells. Hepatocytes and T cells isolated from livers of healthy, untreated HCC, and miR-22-treated HCC mice were subjected to RNA extraction followed by qRT-PCR. Data = mean ± SD (n=3). * p<0.05, ** p<0.01, *** p<0.001 by One-way ANOVA. # Comparison between hepatocytes and T cells by unpaired two-tailed Student's t test.

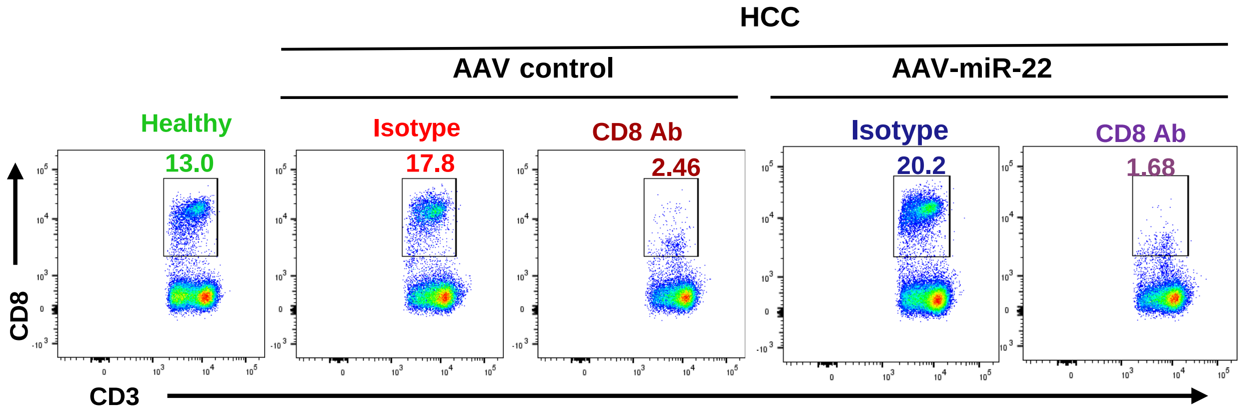

Figure S3. CD8 antibody blockade depletes hepatic CD8$^+$ T cells in HCC mice. Representative flow cytometry plots of CD3$^+$/CD8$^+$ T cells in the livers of healthy, AAV8 control and AAV8-miR-22-treated HCC mice followed by either anti-CD8 mAb or isotype control (n = 4).

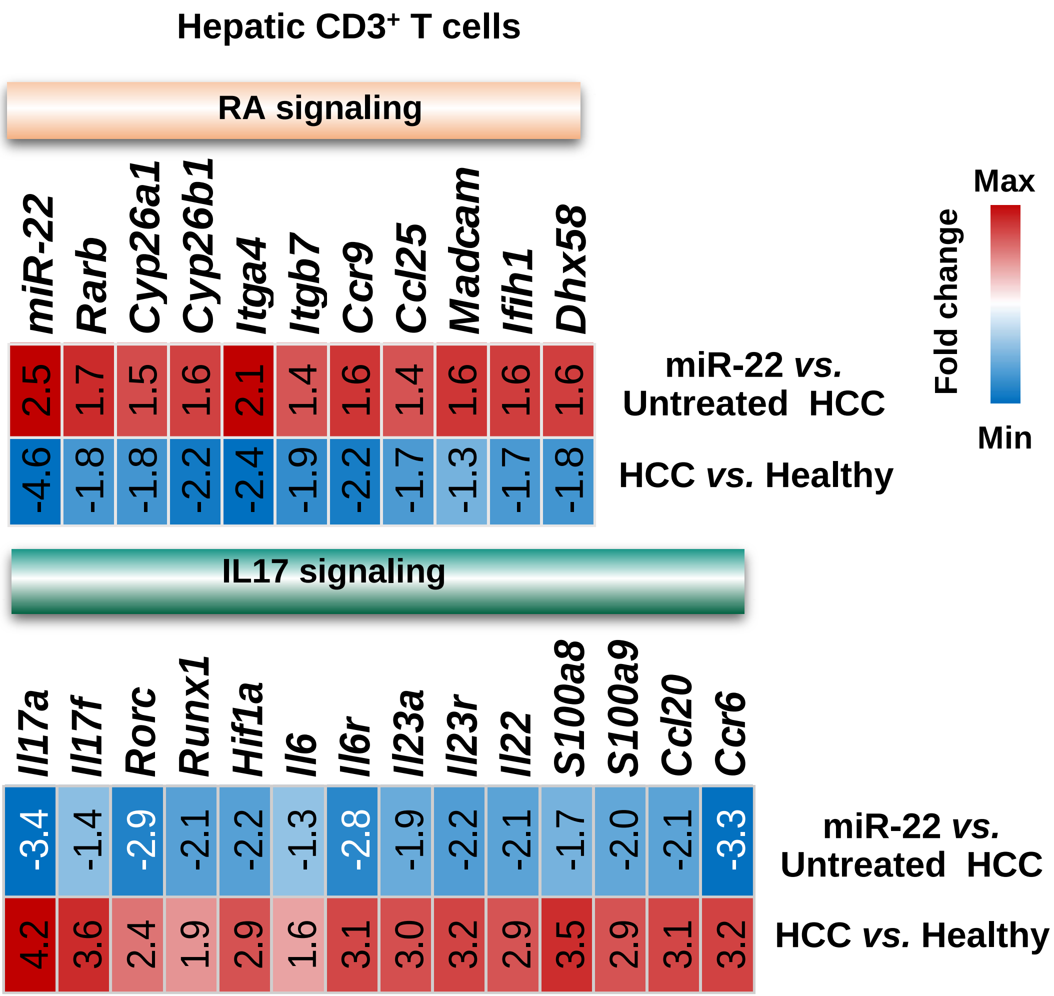

Figure S4. miR-22 increases RA signaling and inhibits IL17 signaling in β-catenin/AKT-driven HCC mice. (A) The fold changes of RA and IL17 signaling-related genes in hepatic T cells were quantified by qRT-PCR and shown in the heatmap. Hepatic T cells isolated from three mice for each group were subjected to RNA extraction followed by qRT-PCR.

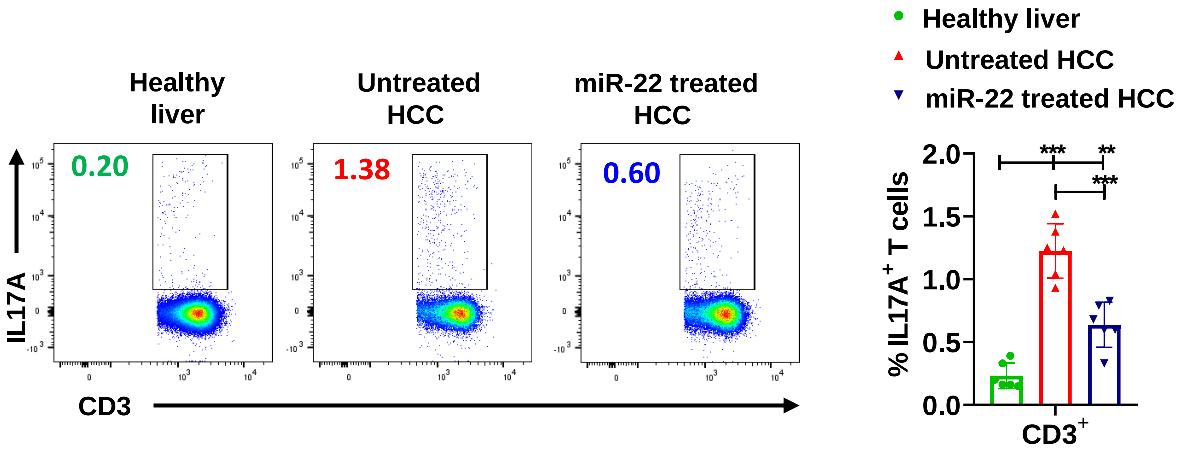

Figure S5. miR-22 treatment reduces IL17-producing T cells. Representative flow cytometry plots and percentage of IL17-producing T cells ($CD3^+IL17A^+$) in the livers of healthy, HCC, and miR-22-treated HCC mice. Data = mean ± SD. ** p<0.01, *** p<0.001 (n = 6 for each group).

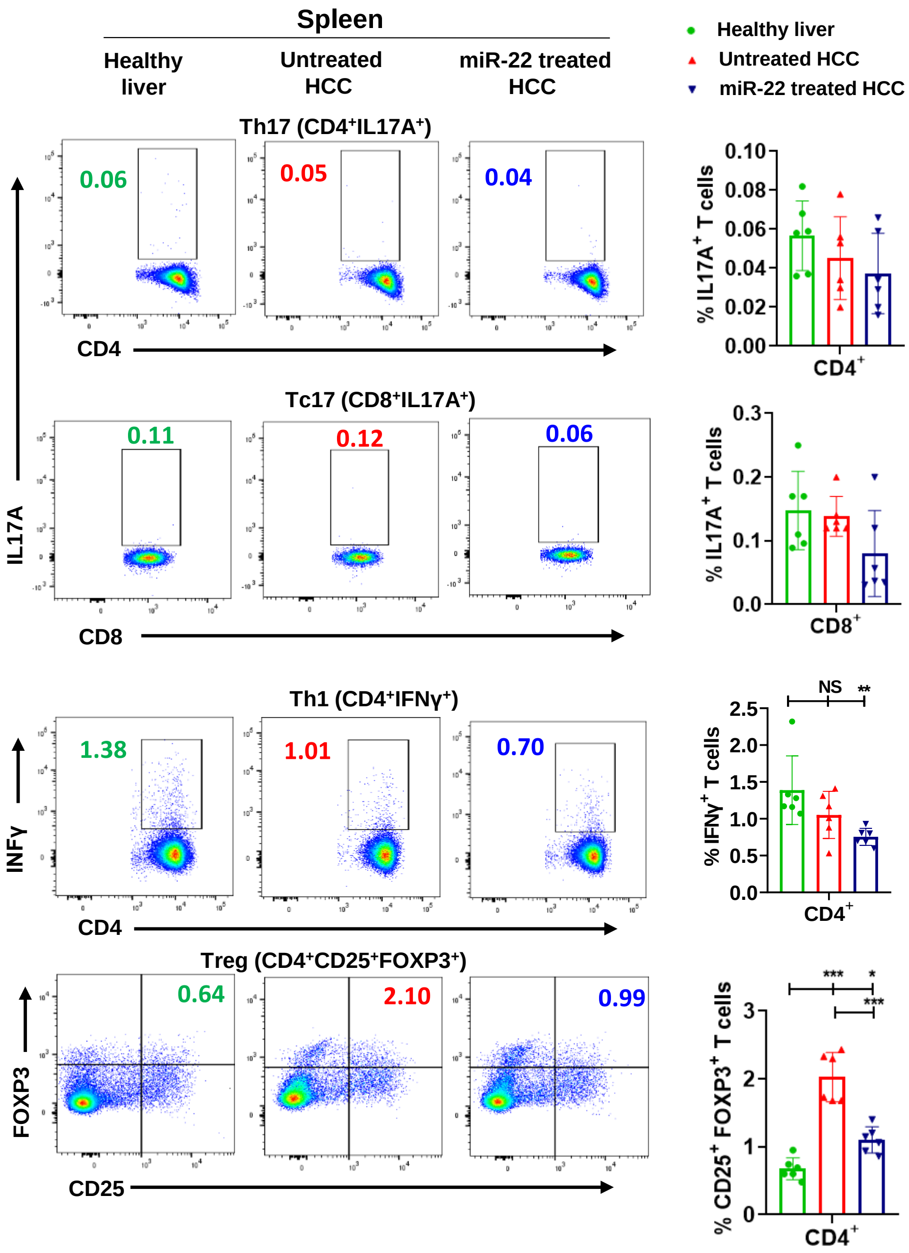

Figure S6. miR-22 treatment reduced Treg and Th1 cells but had no effect on Th17 and Tc17 cells in the HCC splenocytes. Representative flow cytometry plots and percentages of Th17 ($CD4^+IL17A^+$), Tc17 ($CD8^+IL17A^+$), Th1 ($CD4^+IFN\gamma^+$), and Treg ($CD4^+CD25^+FOXP3^+$) T cells from the splenocytes isolated from healthy, untreated HCC, and miR-22-treated HCC mice. Data = mean ± SD. * p<0.05, ** p<0.01, *** p<0.001 by One-way ANOVA (n = 6).

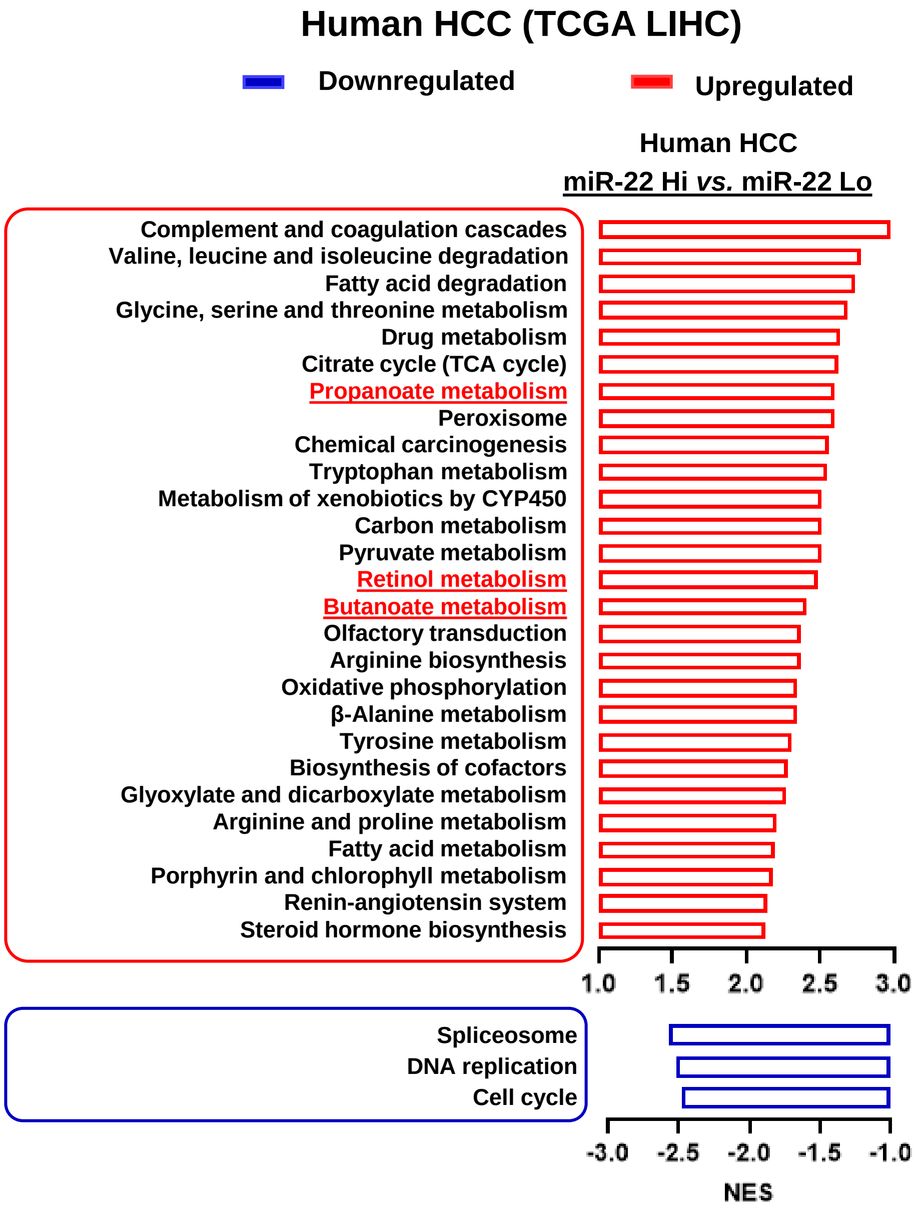

Figure S7. Pathway analysis for the TCGA LIHC human transcriptome profiling based on miR-22 expression levels. Pathways enriched in human HCC by comparing miR-22 high (miR-22 Hi, n=89) *vs*. miR-22 low (miR-22 Lo, n=92) HCC revealed by GSEA based on KEGG gene set. miR-22 inducer signaling (Retinol, Propanoate, and Butanoate metabolism) was underlined and highlighted in red (Upregulated). Normalized Enrichment Score (NES).
