# Supplementary material for: miR-22 gene therapy treats HCC by promoting anti-tumor immunity and enhancing metabolism
Source: Mol Ther. 2023 May 4;31(6):1829–45. doi: 10.1016/j.ymthe.2023.04.019 (PMC10277895; doi:10.1016/j.ymthe.2023.04.019)
Supplement: Document S2. Article plus supplemental information [file mmc2.pdf]

Molecular Therapy
Original Article

miR-22 gene therapy treats HCC by promoting anti-tumor immunity and enhancing metabolism

Ying Hu,[1] Tahereh Setayesh,[1] Farzam Vaziri,[1] Xuesong Wu,[2] Samuel T. Hwang,[2] Xin Chen,[3] and Yu-Jui Yvonne Wan[1]

[1]Department of Pathology and Laboratory Medicine, University of California Davis Health, Sacramento, CA 95817, USA; [2]Department of Dermatology, University of California Davis Health, Sacramento, CA 95817, USA; [3]Cancer Biology Program, University of Hawaii Cancer Center, Honolulu, HI 96813, USA

MicroRNA-22 (miR-22) can be induced by beneficial metabolites that have metabolic and immune effects, including retinoic acids, bile acids, vitamin D_3, and short-chain fatty acids. The tumor suppressor effects of miR-22 have been suggested, but whether miR-22 treats orthotopic hepatocellular carcinoma (HCC) is not established. The role of miR-22 in regulating tumor immunity is also poorly understood. Our data showed that miR-22 delivered by adeno-associated virus serotype 8 effectively treated HCC. Compared with FDA-approved lenvatinib, miR-22 produced better survival outcomes without noticeable toxicity. miR-22 silenced hypoxia-inducible factor 1 (HIF1α) and enhanced retinoic acid signaling in both hepatocytes and T cells. Moreover, miR-22 treatment improved metabolism and reduced inflammation. In the liver, miR-22 reduced the abundance of IL17-producing T cells and inhibited IL17 signaling by reducing the occupancy of HIF1α in the *Rorc* and *Il17a* genes. Conversely, increasing IL17 signaling ameliorated the anti-HCC effect of miR-22. Additionally, miR-22 expanded cytotoxic T cells and reduced regulatory T cells (Treg). Moreover, depleting cytotoxic T cells also abolished the anti-HCC effects of miR-22. In patients, miR-22 high HCC had upregulated metabolic pathways and reduced IL17 pro-inflammatory signaling compared with miR-22 low HCC. Together, miR-22 gene therapy can be a novel option for HCC treatment.

INTRODUCTION

Hepatocellular carcinoma (HCC) is an emerging health burden caused by the increasing levels of obesity.[1,2] However, there is a lack of optimal therapies for HCC.[3] The tyrosine kinase inhibitors including sorafenib, lenvatinib, regorafenib, and cabozantinib have been used as first- or second-line drugs for patients with unresectable HCC.[4,5] However, these agents are associated with considerable toxicities and poor quality of life outcomes, and the survival benefit is limited to a few months.[6] Although immunotherapy has been revolutionary in the management of different types of cancer, the outcomes of HCC treatment are poor.[7,8] Therefore, there is an urgent need to identify novel treatment options for HCC.

MicroRNA-22 (miR-22) is implicated in the development of different cancers, including liver, colorectal, cholangiocarcinoma, prostate, breast, and stomach.[9] miR-22 is generally considered a tumor suppressor but may have oncogenic effects depending on the experimental models.[9–17] For the liver, surprisingly, it has been shown that miR-22 promotes HBV-related HCC development.[18] However, we have uncovered that the expression of miR-22 is induced by beneficial metabolites generated by the microbiota and the host within the gut-liver axis.[19,20] These metabolites include retinoic acid (RA), short-chain fatty acids (SCFAs), bile acids (BAs), and vitamin D_3, which have metabolic and anti-cancer benefits.[19–21] Signaling regulated by these chemicals and their receptors is usually compromised during dysbiosis-associated inflammation or liver tumorigenesis.[22–26] In the past, we have shown that miR-22 has anti-cancer effects in the colon by exporting the nuclear receptors RARβ and NUR77 to the cytosol and targeting mitochondria to induce apoptosis.[20] Other mechanisms include silencing of cyclin A2 (*CCNA2*) and inhibition of cancer cell migration.[19,27] miR-22 also silences fibroblast growth factor 21 and its receptor FGFR1 to inhibit metabolism-driven growth and proliferation controlled by ERK1/2 signaling.[28] Further, miR-22 levels are inversely correlated with serum α-fetoprotein (AFP) levels, suggesting its tumor-suppressive role in HCC.[29] However, whether miR-22 treats HCC has not been studied in orthotopic preclinical models.

The role of miR-22 in the modulation of the tumor immune microenvironment is less understood. Recent studies showed that miR-22 downregulates checkpoint molecule programmed death-ligand 1, which inhibits T cell-mediated immune responses in colon cancer.[30] miR-22 is highly expressed in regulatory T cells (Treg) from multiple sclerosis patients.[31] Additionally, miR-22 indirectly regulates the T helper 17 (Th17) responses by controlling the activation of myeloid dendritic cells in an emphysema mouse model.[32] However, it is still

Received 26 February 2023; accepted 28 April 2023;
https://doi.org/10.1016/j.ymthe.2023.04.019.

Correspondence: Yu-Jui Yvonne Wan, PhD, Department of Pathology and Laboratory Medicine, University of California Davis Health, Room 3400B, 4645 2nd Ave, Research Building III, Sacramento, CA 95817, USA.
E-mail: yjywan@ucdavis.edu

This is an open access article under the CC BY license (http://creativecommons.org/licenses/by/4.0/).

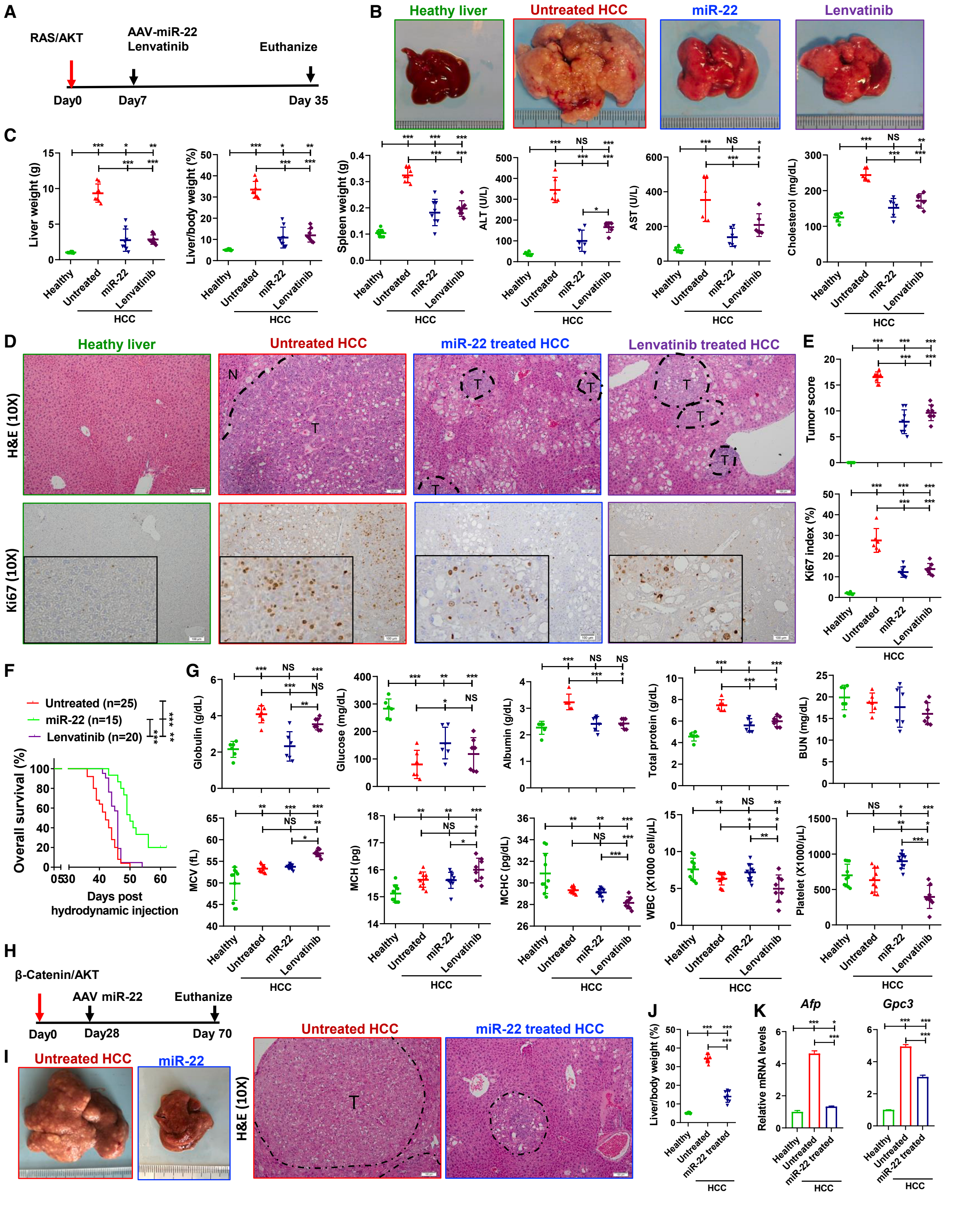

(legend on next page)

unclear how miR-22 regulated the functions of T cells. Thus, the current study examined the anti-HCC effects of miR-22 particularly focusing on its immunomodulatory functions in T cells using orthotopic mouse HCC models.

Our data revealed that miR-22 treated HCC and prolonged survival time. Compared with lenvatinib treatment, miR-22 therapy provided a longer survival time without detectable toxicity. Moreover, miR-22 treatment effectively silenced hypoxia-inducible factor 1 (HIF1α) and enhanced RA signaling in both hepatocytes and T cells, which improved metabolism and anti-tumor immunity. The miR-22-targeted metabolism and inflammation signaling observed were human relevant, as indicated by comparing miR-22 high vs. low human HCC. miR-22 gene therapy, targeting both hepatocytes and T cells, may become a novel option for HCC treatment.

RESULTS

miR-22 treats HCC

Activation of AKT and the neuroblastoma RAS viral oncogene homolog (RAS) is frequently observed in patients with HCC.[6,33] To examine the anti-HCC effect of miR-22, RAS/AKT-induced HCC female mice were treated with either AAV8-miR-22 (miR-22 treated) or AAV8 control (untreated). Lenvatinib, an FDA-approved HCC drug, was used for comparison (Figure 1A). In untreated control groups, 5 weeks post oncogene injection, the liver-to-body weight (L/B) ratio reached 33.5%, 8-fold higher than that in healthy mice. Tumors accounted for more than 90% of liver sections. Treatment with miR-22 and lenvatinib reduced the L/B ratio to 10.9% and 12.0%, respectively (Figures 1B and 1C). The effectiveness of both treatments was confirmed by histological evaluation (Figures 1D and 1E). miR-22 and lenvatinib reduced the serum alanine transaminase (ALT), aspartate aminotransferase (AST), and cholesterol levels, indicating improved liver function (Figures 1B and 1C). Additionally, miR-22 and lenvatinib treatment markedly reduced the number of Ki67-positive cells (Figures 1D and 1E). Splenomegaly is typically found in HCC patients and mice, likely due to increased infiltration of foreign cells in the spleen[34]; both miR-22 and lenvatinib reduced spleen size significantly (Figure 1C).

The anti-HCC effect of miR-22 was also confirmed in male mice. miR-22 treatment significantly reduced tumor burden as indicated by significant reductions in the liver weight and L/B ratio in male HCC mice (Figures S1A–S1C). In addition, terminal deoxynucleotidyl transferase dUTP nick end-labeling (TUNEL) analysis that showed apoptosis of HCC cells was demonstrated in miR-22-treated HCC (Figure S1D).

miR-22-treated mice have longer survival time without toxicity compared with lenvatinib-treated mice

In the RAS/AKT-HCC female mice, the median survival time of the HCC mice was 42 days. Two mice treated with miR-22 remained alive 60 days post oncogene injection. Excluding these two, miR-22 treatment was found to extend the survival time to 50 days. In contrast, the median survival time of the lenvatinib-treated group was 46 days (Figure 1F).

Patients with HCC commonly have elevated serum globulin levels, which was also observed in our mouse models.[35] miR-22 treatment reduced serum globulin levels; however lenvatinib did not. Moreover, HCC mice had reduced serum glucose levels, which are also observed in patients with HCC as indicators of hypoglycemia and poor prognosis.[36] Serum glucose levels were also reduced in response to miR-22 treatment but not lenvatinib. Both miR-22 and lenvatinib reversed the elevation in total protein and albumin levels. Blood urea nitrogen (BUN) levels were not affected by HCC or either treatment, suggesting no renal toxicity (Figure 1G).

Complete blood counts indicated that HCC mice had macrocytic anemia, as evidenced by increased mean corpuscular volume and mean corpuscular hemoglobin, as well as reduced mean corpuscular hemoglobin concentration (Figure 1G). Macrocytic anemia is commonly observed in HCC.[37] Lenvatinib exacerbated this condition, whereas miR-22 did not. Furthermore, lenvatinib-treated mice had the lowest white blood cell and platelet counts. In contrast, miR-22 treatment increased the levels of both (Figure 1G).

miR-22 treats β-catenin-positive HCC.

β-Catenin activation promotes immune escape.[38] β-Catenin-positive HCC, accounting for 50% of human HCC cases, is resistant to anti-PD-1 treatment.[38] The anti-cancer effect of miR-22 was studied in β-catenin/AKT-induced female mouse HCC. miR-22 treatment was initiated 4 weeks after oncogene delivery (Figure 1H) and significantly reduced the L/B ratio compared with that in untreated mice (Figure 1J). The gross morphology and histology are shown in Figure 1I. Furthermore, miR-22 reduced the expression of the HCC markers *Afp* and *Gpc3* (Figure 1K).

miR-22 treatment restores hepatic metabolism and reduces inflammatory signaling

To uncover the pathways altered due to HCC development and the response to miR-22 treatment, RNA sequencing was performed using the livers derived from female HCC mice, followed by gene set

Figure 1. miR-22 treats HCC and prolongs survival in female HCC mice

(A) Study design for miR-22 and lenvatinib treatment in RAS/AKT-induced HCC model, (B) representative liver morphology, (C) liver weight, L/B ratio, spleen weight, serum ALT, AST, and cholesterol levels, and (D and E) H&E-stained liver sections and Ki67 IHC staining. The cellularity of the proliferating cells is seen at high magnification (insets). The tumor score was quantitively evaluated, which is detailed in Table S3. The percentage of Ki67-positive cells was determined in five random x10 microscopic fields for each section. Scale bar, 100 μm. (F) Kaplan-Meier survival curves of overall survival of three groups (n = 15–25/group). (G) Toxicology for the studied groups. (H) Study design of miR-22 treatment in β-catenin/AKT-induced HCC model. (I) Representative liver morphology and H&E-stained liver sections (scale bar, 100 μm), (J) L/B ratio, and (K) hepatic mRNA levels of HCC markers *Afp* and *Gpc3* for studied group. Data represent mean ± SD (n = 6–8/group for B, C, E, G, I, J, and K). *p < 0.05, **p < 0.01, ***p < 0.001 by one-way ANOVA (C, E, F, G, J, and K).

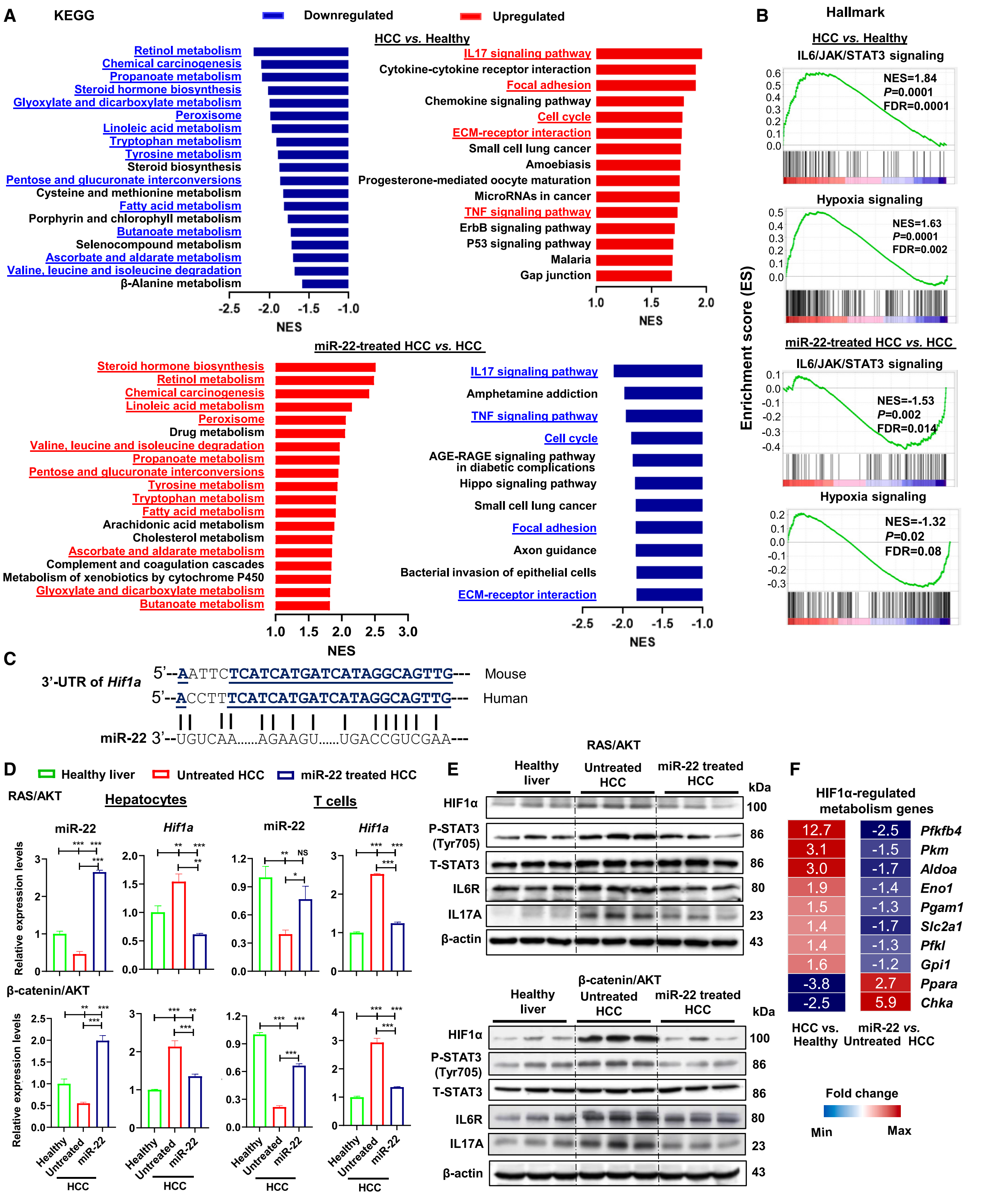

HCC vs. Healthy	miR-22 vs. Untreated HCC	Gene
12.7	-2.5	*Pfkfb4*
3.1	-1.5	*Pkm*
3.0	-1.7	*Aldoa*
1.9	-1.4	*Eno1*
1.5	-1.3	*Pgam1*
1.4	-1.7	*Slc2a1*
1.4	-1.3	*Pfkl*
1.6	-1.2	*Gpi1*
-3.8	2.7	*Ppara*
-2.5	5.9	*Chka*

(legend on next page)

enrichment analysis (GSEA) using the Kyoto Encyclopedia of Genes and Genomes (KEGG) gene set. Compared with healthy livers, downregulated pathways found in HCC were related to the metabolism of retinol (RA), propanoate and butanoate (SCFAs), fatty acids, steroid hormones (BAs), peroxisomes (functions of BA), tryptophan, sugar, and amino acids. Among these, many are miR-22 inducers (pathways in parentheses). Importantly, miR-22 treatment induced 14 of the 19 downregulated pathways (Figure 2A).

The upregulated pathways in HCC were related to inflammation, including IL17 signaling, cytokine-cytokine receptor interaction, extracellular matrix (ECM)-receptor interaction, and chemokine signaling. Additionally, cell cycle, p53 signaling, and focal adhesion pathways were upregulated in HCC. In contrast, miR-22 treatment reduced IL17 signaling, TNF signaling, cell cycle, and ECM-receptor interaction (blue, Figure 2A, lower panel). Increased IL17 signaling in HCC was accompanied by enriched IL6/JAK/STAT3, crucial for IL17 transcription and Th17 differentiation (Figure 2B). Increased hypoxia signaling observed in HCC was also inhibited by miR-22 treatment (Figure 2B). Overall, miR-22 treatment improved metabolism, inhibited inflammation, and reduced hypoxia signaling.

Hepatocytes and T cells isolated from HCC have reduced miR-22 and increased HIF1α, which are reversed by miR-22 treatment

Hypoxia induces HIF1α, a master transcription factor that regulates metabolism and inflammation.[39,40] HIF1α is an miR-22 target[41]; an miR-22 homolog motif is shown in the 3′ untranslated region (3′ UTR) of the *Hif1a* gene (Figure 2C). Thus, the expression levels of miR-22 and HIF1α were quantified in the liver, as well as in isolated hepatocytes and T cells, using western blotting or RT-PCR, respectively. The purity of isolated cells was validated by the expression of cell-type-specific markers (Figure S2). In both HCC hepatocytes and T cells, reduced miR-22 and increased *Hif1a* expression were observed. miR-22 treatment reversed the expression pattern found in both the RAS/AKT- and β-catenin/AKT models (Figure 2D). Taken together, miR-22 treatment impacted both hepatocytes and T cells.

Western blotting validated these findings at the protein level. In both RAS/AKT- and β-catenin/AKT HCC models, untreated HCC had increased HIF1α, activated STAT3, and increased inflammatory signaling of IL6R and IL17A, which were all reduced by miR-22 (Figure 2E).

HIF1α has known metabolic roles, and the expression of HIF1α-regulated metabolic genes was altered due to HCC development and the response to miR-22 treatment. HCC increased the expression of glycolysis-related genes including *Pfkfb4*, *Pkm*, *Aldoa*, *Eno1*, *Pgam1*, *Slc2a1*, *Pfkl*, and *Gpi1*.[42] Whereas, two known HIF1α-suppressed lipid metabolism-related genes, *Ppara* (fatty acid oxidation) and *Chka* (phospholipid synthesis), were reduced in HCC livers.[43,44] Upon miR-22 treatment, these changes were reversed (Figure 2F).

The anti-HCC effect of miR-22 is cytotoxic T cell dependent

Hypoxia and HIF1α can induce tumor cells to become resistant to cytotoxic T cells.[45] Flow cytometry was performed to investigate the contribution of cytotoxic T cells to the anti-HCC effect of miR-22. Treatment with miR-22 increased $CD8^{+}IFN\gamma^{+}/CD8^{+}CD107A^{+}$ T cells, indicating activation of cytotoxic $CD8^{+}$ T cells (Figure 3A). In parallel, miR-22 increased $CD8^{+}$ effector memory T cells, which are known to enhance the therapeutic effects of anti-PD-1 in head and neck cancers (Figure 3A).[46] CD8 blockade was then performed (Figure 3B). While anti-CD8 antibody did not affect the L/B ratio and tumor development, CD8 blockade inhibited the anti-HCC effects of miR-22 (Figures 3C and 3D). Flow cytometric analysis confirmed that the anti-CD8 antibody significantly reduced the number of $CD8^{+}$ T cells in the liver (Figures 3E and S3).

miR-22 activates cytotoxic T cells and induces apoptosis of HCC cells

The essential role of cytotoxic $CD8^{+}$ T cells in miR-22 treatment was further examined. T cells isolated from livers of healthy, HCC, and miR-22-treated HCC mice were co-cultured with mouse Hepa1-6 HCC cells for 36 h, followed by an apoptosis assay. The data showed that T cells from miR-22-treated mice induced the highest apoptotic rate (Figure 3F), and the culture supernatant also had the highest levels of IFNγ and granzyme B. Compared with healthy controls, HCC T cells produced elevated levels of IL17A and IL6, which were reduced by miR-22 treatment (Figure 3G).

miR-22 increases RA and reduces Th17/Treg signaling in hepatic T cells

We have previously shown that RA via RARβ induces miR-22, which in turn induces RARβ by silencing HDCAs. Thus, RARβ is involved in both upstream and downstream signaling of miR-22.[20] This positive regulatory loop ensures sustained RARβ expression. RA, via RARβ, has profound effects on the inhibition of Th17 cell differentiation.[47] Hepatic transcriptomics revealed an improved retinol metabolism in response to miR-22 treatment. Therefore, we investigated RA signaling in hepatic T cells.

Figure 2. miR-22 treatment restores metabolic programs and reduces inflammatory signaling accompanied by reduced HIF1α expression in the liver, hepatocytes, and T cells

(A) Pathways enriched due to HCC formation or miR-22 treatment revealed by GSEA based on KEGG gene sets. miR-22-reversed pathways are underlined and highlighted in red (upregulated) or blue (downregulated). NES, normalized enrichment score. (B) Enriched IL6/JAK/STAT3 and hypoxia signaling by comparing HCC vs. healthy livers or miR-22 treated vs. untreated HCC as demonstrated by GSEA based on hallmark gene sets. (C) Human and mouse miR-22 have conserved sequences, which partially pair with the 3′ UTR of the human and mouse *Hif1a* gene. (D) The level of miR-22 and HIF1α in hepatocytes and T cells isolated from livers of healthy, HCC, and miR-22-treated HCC mice (n = 3). (E) The levels of indicated proteins in the HIF1α/IL6/STAT3/IL17 axis were determined by western blot (n = 3). (F) The fold changes of HIF1α-regulated metabolism-related genes are shown in the heatmap based on RNA-seq data. Data represent mean ± SD. *$p < 0.05$, **$p < 0.01$, ***$p < 0.001$ by one-way ANOVA (D).

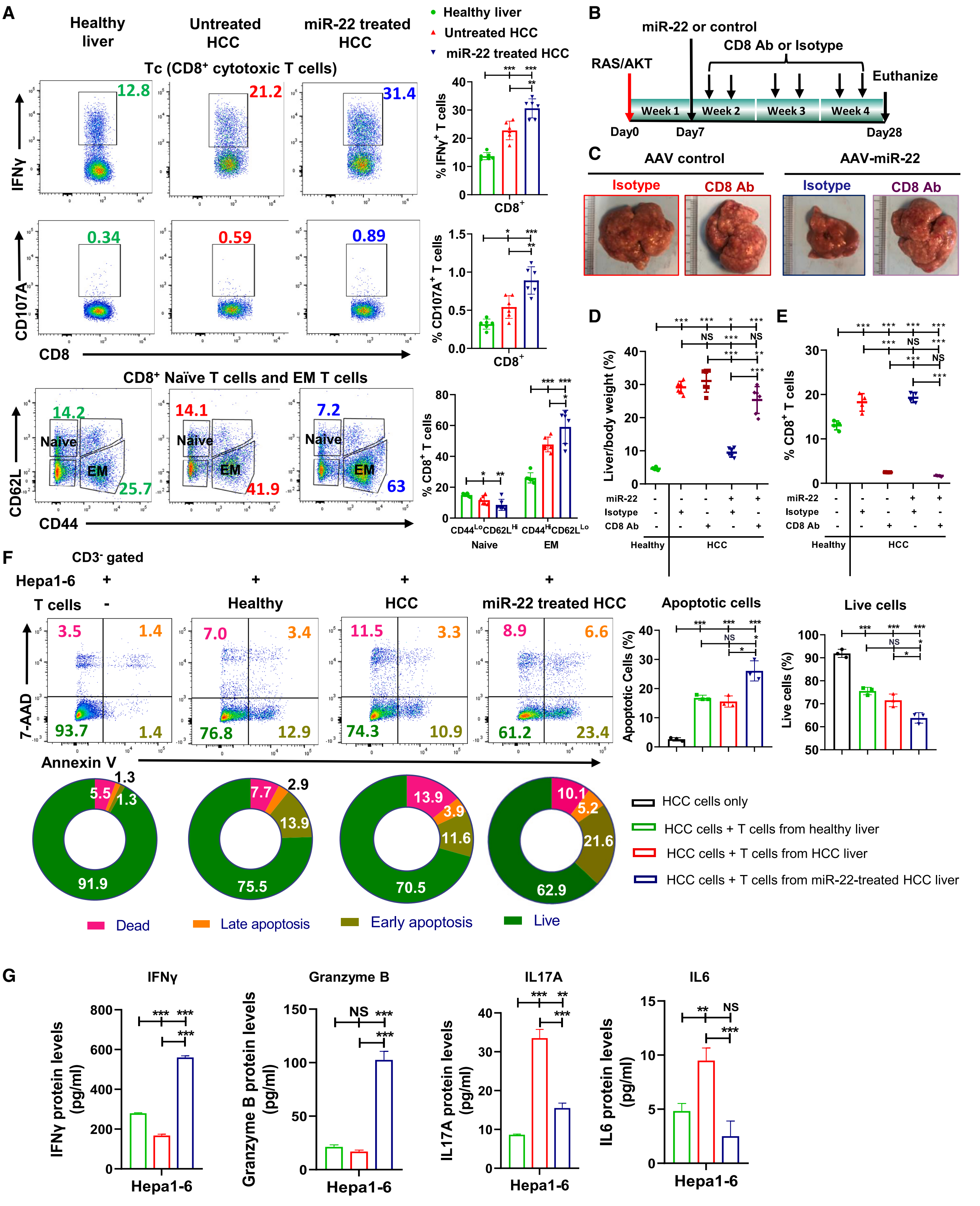

(legend on next page)

Consistent with the bulk liver transcriptomic data, RA signaling was reduced in HCC T cells, which was reversed by miR-22 treatment. This was evident by the induction of genes encoding RARβ, as well as RA oxidation enzymes CYP26A1 and CYP26B1. Moreover, miR-22-treated T cells showed increased expression of RA-regulated genes that control immunity,[48] including the gut-homing genes *Itga4*, *Itgb7*, *Ccr9*, *Ccl25*, and *Madcam1*. Other miR-22-induced RA signaling genes, *Ifih1* and *Dhx58,* can recruit T cells to inflammation sites and enhance $CD8^+$ T cell survival (Figure 4A).[49]

IL17 signaling genes significantly upregulated in HCC included *Il17a*/f, *Il17a* transcription regulators *Rorc, Runx1,* and *Hif1a*, upstream regulatory cytokines *Il6*, *Il23a*, and their receptors, as well as Th17 cell downstream signaling *Il22*, *Ccl20*, *Ccr6*, *S100a8*, and *S100a9*. miR-22 treatment suppressed all these IL17 signaling genes (Figure 4A). Tregs have immunosuppressive functions in cancer. Many Treg genes were elevated in HCC T cells but repressed by miR-22 treatment, including *Il2*, surface markers *Il2ra*, *Nt5e*, *Lag3*, *Nrp1*, *Ctla4*, and downstream factors *Lgals1*, *Il10*, *Il12a*, and *Il35b* (Figure 4A).

Similarly, in β-catenin-positive HCC, qRT-PCR data showed that many genes involved in RA signaling were downregulated in HCC T cells but induced by miR-22. In contrast, IL17 signaling-associated genes were induced in HCC and reduced in miR-22-treated T cells (Figure S4).

miR-22 treatment reduces IL17-producing T cells as well as Treg

Flow cytometry was performed to validate these findings. Compared with healthy livers, HCC had increased $CD3^+IL17A^+$ T cells, which miR-22 treatment then reduced (Figure S5). The $CD4^+$ subsets (Th17, Treg, Th1) and Tc17 ($CD8^+IL17A^+$) T cells were increased in HCC. While miR-22 treatment reduced Th17 and Treg cells, it did not affect Th1 and Tc17 cells (Figure 4B). Taken together, miR-22 suppressed the expansion of IL17-producing cells and Tregs, which likely favored cytotoxic $CD8^+$ T cell activation.[50]

HCC mice spleens had increased Treg cells, whereas Th17, Tc17, and Th1 cells were similar to healthy mice. In contrast, miR-22 treatment reduced Treg and Th1 cells but had no effect on Th17 and Tc17 cells (Figure S6). Thus, Th17 expansion in HCC and its reduction by miR-22 were tumor specific.

miR-22 silences HIF1α and inhibits *Il17a* expression by reducing the recruitment of HIF1α/RORγT/STAT3

There are two RORγT and two STAT3 binding sites in the regulatory region of the *Il17a* gene (Figure 4C).[51,52] Moreover, the *Rorc* promoter contains two hypoxia response elements (HREs).[51] The occupancy of the transcription factors HIF1α, RORγT, and STAT3 was examined by chromatin immunoprecipitation-qPCR (ChIP-qPCR) in HCC T cells. In untreated HCC T cells, HIF1α bound to the two HREs located in the *Rorc* promoter, while miR-22-treated T cells showed reduced binding. Similarly, miR-22-treated HCC T cells showed reduced recruitment of HIF1α, RORγT, and STAT3 to the *Il17a* promoter (Figure 4C). These results showed that miR-22 silenced the *Il17a* gene, leading to Th17 cell reduction by decreasing the recruitment of RORγT/HIF1α/STAT3 to *Il17a* promoter in T cells.

Overexpression of IL23/IL17 attenuates the anti-HCC effect of miR-22

To further establish that IL17A signaling inhibition contributes to the anti-HCC effect of miR-22, a low dose of IL23 minicircle DNA was introduced to boost IL17 (Figure 5A).[53] The results showed inducing IL17 attenuated the anti-HCC effect of miR-22, as evidenced by tumor load and histology (Figures 5B–5D). Furthermore, in HCC T cells, IL23 increased *Il23, Il17a*, *Il17f*, *S100a8*, *S100a9*, *Ccr6*, and *Ccl20* levels (Figure 5E). IL23 overexpression prevented miR-22 reduction of HCC markers *Afp*, *Gpc3*, *Cd133*, and *Ccna2* (Figure 5F). Together, reduced IL17 signaling contributed to the anti-HCC effects of miR-22.

The similarity between human and mouse HCC based on miR-22 expression

Based on miR-22 expression, the TCGA LIHC dataset was grouped into miR-22 high (miR-22 Hi, n = 89) and miR-22 low (miR-22 Lo, n = 92) HCC. Comparing the two groups, 30 pathways (27 upregulated and three downregulated pathways) were significantly changed (Figure S7). In mouse HCC, miR-22 treatment enriched 30 pathways (19 upregulated and 11 downregulated; Figure 2A); 15 pathways overlapped with human pathways by comparing miR-22 high vs. low human HCC (Figure 6A). Human miR-22 Hi HCC or miR-22-treated mouse HCC consistently showed increased retinol, propanoate, butanoate, and tryptophan metabolism, improved steroid biosynthesis, as well as glycolysis and detoxification, but reduced cell cycle signaling. This congruence suggests human relevance for the mice data.

Based on the KEGG pathways, the expression of genes involved in retinol metabolism and IL17 signaling was analyzed in miR-22 Hi vs. Lo HCC (Figure 6B). For retinol metabolism, 30/32 related transcripts showed higher levels in miR-22 Hi HCC than in miR-22 Lo HCC. In addition, miR-22 Hi HCC had reduced IL17 signaling in 20/27 transcripts. Fold changes with significance levels are indicated in Figure 6B.

Figure 3. The anti-HCC effect of miR-22 is cytotoxic T cell dependent, and miR-22 activates cytotoxic T cells to induce apoptosis of HCC cells
(A) Representative flow cytometry plots and percentage of $CD8^+IFN\gamma^+$, $CD8^+CD107A^+$, and $CD8^+$ naive and EM T cells. Hepatic lymphocytes were isolated from livers of healthy, HCC, and miR-22-treated HCC mice followed by flow cytometry. (n = 6). (B) Study design of anti-CD8 antibody blockade. (C) Representative liver morphology, (D) L/B ratio (n = 6), and (E) percentage of $CD8^+$ T cells measured by flow cytometry in studied groups (n = 4). (F) Representative flow cytometry plots of Annexin V/7-AAD staining and apoptosis rates of mouse HCC Hepa1-6 cells co-cultured with hepatic T cells. Hepatic-isolated T cells from healthy livers, HCC, and miR-22-treated HCC were co-cultured with Hepa1-6 at a 1:1 ratio for 36 h. (G) The concentrations of IFNγ, granzyme B, IL17A, and IL6, and in the supernatant were quantified by ELISA. Data are representative of two independent experiments (F and G). Data represent mean ± SD. $^*p < 0.05$, $^{**}p < 0.01$, $^{***}p < 0.001$ by one-way ANOVA (A, D, E, F, and G).

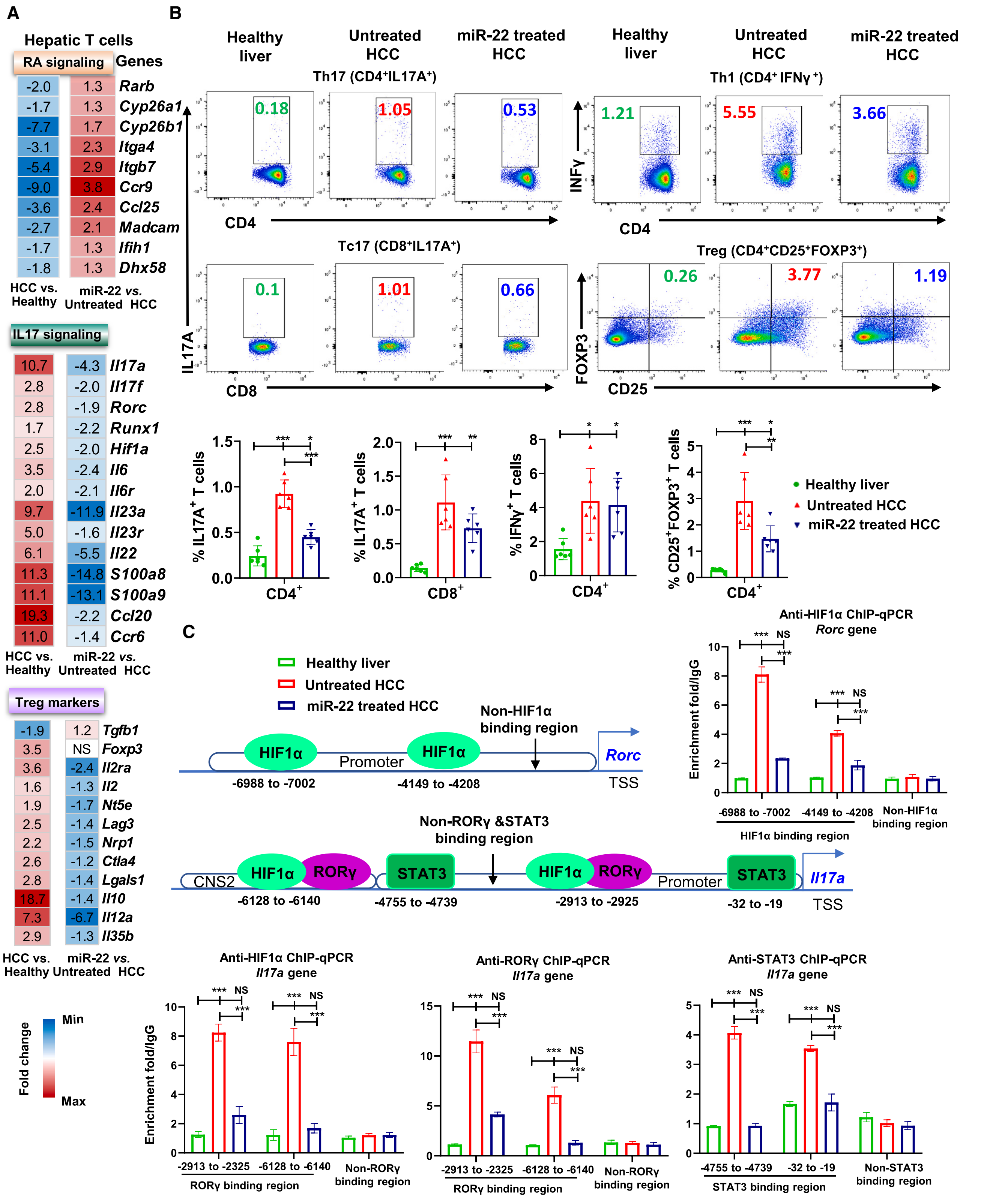

(legend on next page)

Reduced miR-22 in human HCC predicts poor survival outcomes

miR-22 expression in human HCC was analyzed using the TCGA LIHC dataset. miR-22 levels were significantly lower in 379 HCC specimens compared with the 59 healthy livers. Additionally, miR-22 levels were much lower in stage III and IV HCC (90 cases) than in stage I (172 cases) (Figure 6C). Furthermore, survival analyses revealed that miR-22 levels positively correlated with overall survival, disease-specific survival, disease-free interval, and progression-free interval (Figure 6D). In summary, reduced miR-22 expression predicted poor survival outcomes in patients with HCC.

DISCUSSION

The current study revealed that miR-22 could be a new therapeutic target for HCC treatment. miR-22 gene therapy was effective and prolonged survival without causing detectable toxicity. The current study is also the first to reveal the metabolic and anti-tumor immunity roles of miR-22 in the liver; miR-22 targeted both hepatocytes and T cells by silencing HIF1α and increasing RA signaling, both of which have metabolic and anti-inflammatory effects.

Our data revealed that miR-22 had profound effects on reducing inflammation. In T cells, miR-22 affected *Il17a* expression at multiple levels: (1) miR-22 reduced HIF1α and its occupancy of the *Rorc* gene, leading to reduced expression. (2) miR-22 diminished the recruitment of HIF1α and RORγT to *Il17a* and reduced IL17 expression. (3) miR-22 inhibited IL6/IL23 signaling, deactivated STAT3, and reduced its recruitment to *Il17a*, suppressing IL17 levels. Additionally, miR-22 activated cytotoxic T cells, induced apoptosis in tumor cells, and suppressed Treg cells, which can inhibit the activation of anti-tumor effector cells, leading to tumor immune escape.[54] These results are summarized in Figure 7.

RA self-regulates via RARβ binding to induce *Rarb* expression.[55] RA also increases RARβ via the induction of miR-22.[20] Thus, multiple pathways induce and sustain RA signaling, signifying its importance. Increasing RA signaling has several benefits. RA and its receptors, including RXRα, facilitate lipid metabolism by dimerizing with FXR and PPARα. RA also inhibits the production of Th17 cells by blocking IL23/IL6 signaling and reducing *Rorc* expression.[47,56] In line with this, our data revealed that restoration of RA signaling by miR-22 was accompanied by reduced *Rorc, Runx1*, *Il6, Il6r*, *Il23*, and *Il23r* mRNA in T cells. Furthermore, IL23 overexpression boosted IL17 signaling and attenuated the anti-HCC effects of miR-22, indicating that miR-22 treatment was partly mediated by the inhibition of inflammatory IL17A signaling. Thus, miR-22 and RA mutually regulate one another. Whether miR-22-suppressed IL17 signaling is RA dependent would be of interest for future studies.

Patients with HCC have a high frequency of Tregs, which is correlated with poor prognosis and reduced survival.[57] Depleting these tumor-infiltrating Treg cells improves immunotherapy of HCC.[58] Our data revealed that miR-22 reduced Tregs and the expression of Treg marker genes in HCC cells. However, RA plays a role in Treg expansion, and this mechanism is partially mediated by RARα-mediated TGFβ and FOXP3 induction.[47,51] Whether miR-22-regulated RA signaling affects Treg regulation in the HCC environment remains to be addressed.

Interestingly, the synthesis of RA in intestinal dendritic cells is controlled by aldehyde dehydrogenase ALDH1A, whose expression is induced by HDAC inhibitors, such as butyric acid and propionic acid, which are miR-22 inducers.[20,47] Thus, there are interactive effects between SCFAs and RA signaling. Our transcriptomic data revealed that the metabolic pathways for SCFAs and retinol were both reduced in HCC but induced in response to miR-22 treatment. The effect of miR-22 on the regulation of the gut microbiome remains to be elucidated. These data suggest that the intertwined signaling pathways found in the gut affect liver health. Targeting these pathways in the gut-liver axis may provide novel therapeutic options for treating HCC.

MATERIALS AND METHODS

Mice and tumor models

Male and female 6-week-old FVB/N mice were obtained from Jackson Laboratory (Sacramento, CA, USA). Liver tumors were produced via sleeping beauty transposon (SB)-mediated hydrodynamic injection using the plasmids pT3-EF1α-HA-myr-AKT, pT3-EF1α-N90-β-catenin, pT/Caggs-Nras-v12, and pCMV-SB11 as previously described.[59] Briefly, oncogene plasmids and SB were diluted in 2 mL saline and injected into the mouse tail vein within 5–7 s. Mice were housed, fed, and monitored according to protocols approved by the Institutional Animal Care and Use Committee of the University of California, Davis (Sacramento, CA, USA).

Drug administration

miR-22 was delivered by adeno-associated virus, serotype 8 (AAV8, Applied Biological Materials, Richmond, BC, Canada). One dose (5 x 10^{12} GC/kg) of AAV8-miR-22 or AAV8 blank control was injected intravenously. Lenvatinib (10 mg/kg/day, MedChemExpress, Monmouth Junction, NJ, USA) or saline was administered via oral gavage. The treatment timeline for each experiment is presented in the figures.

Figure 4. miR-22 suppresses IL17 signaling in the T cells by reducing the recruitment of HIF1α/RORγT/STAT3 in the *Il17a* promoter

(A) The fold changes of RA signaling and Th17/Treg-related genes in hepatic T cells were quantified by RT-PCR and are shown in the heatmap. Hepatic T cells were isolated from livers of healthy, HCC, and miR-22-treated HCC mice followed by flow cytometry (n = 3). (B) Representative flow cytometry plots and percentage of Th17 ($CD4^+IL17A^+$), Tc17 ($CD8^+IL17A^+$), Th1 ($CD4^+IFN\gamma^+$), and Treg ($CD4^+CD25^+FOXP3^+$) T cells in studied groups (n = 6). (C) ChIP-qPCR using anti-HIF1α, anti-RORγt, and anti-STAT3 antibodies in hepatic T cells. Hepatic T cells that were isolated from three mice for each studied group were subjected to ChIP assay. The primers for amplifying non-binding regions were used as a negative control. The regulatory regions of the *Il17a* and *Rorc* genes are shown with the binding locations of the indicated proteins. The numbers are relative to the transcription start site. Binding enrichment was expressed relative to the IgG-negative control. CNS, conserved non-coding sequence. Data represent mean ± SD, $^{**}p < 0.01$; $^{***}p < 0.001$ by one-way ANOVA (B and C).

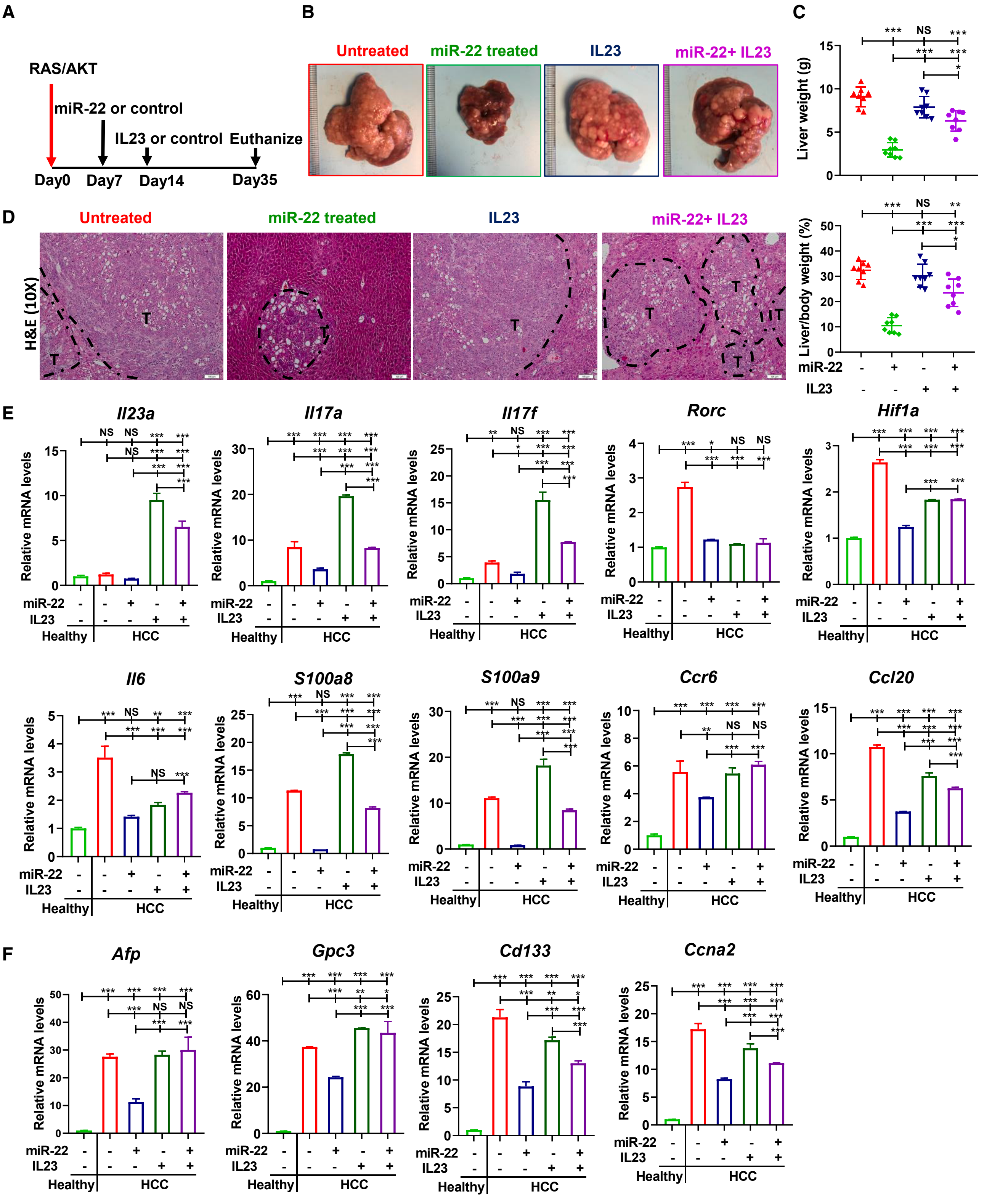

(legend on next page)

Hepatic lymphocyte isolation and T cell enrichment

Hepatic lymphocytes were isolated from livers of healthy, HCC, and miR-22-treated HCC mice using previously published methods.[60] In detail, the livers were mechanically dissociated followed by digestion with Liberase (0.05 mg/mL, Roche Diagnostics, Basel, Switzerland) at 37°C for 30 min. The digested specimens were filtered through a 70-μm cell strainer. Hepatocytes were removed by sequential centrifugation at 50 x *g* (5 min, 4°C). The supernatants containing nonparenchymal cells (NPCs) were collected by centrifugation at 500 x *g* for 5 min at 4°C. The NPCs were further fractionated by Percoll (GE Healthcare, Little Chalfont, United Kingdom) density gradient (70%/30%) centrifugation at 690 x *g* for 12 min at room temperature. Red blood cells were removed by incubating in RBC lysis buffer (Thermo Fisher Scientific, Waltham, MA, USA) for 5 min at room temperature. After washing with PBS, NPCs were pelleted by centrifugation at 300 x *g* for 5 min. $CD3^+$ T cells were further purified using a MojoSort mouse $CD3^+$ T cell Isolation Kit (Biolegend, San Diego, California, USA) followed by RNA extraction, ChIP, or co-culture. The purity of $CD3^+$ T cells was >90%.

Cloning and packaging of miR-22 overexpressed adeno-associated virus

Mature mmu-miR-22-3p (ID: MIMAT0000531, https://www.mirbase.org/cgi-bin/mature.pl?mature_acc=MIMAT0000531) was cloned into AAV8 plasmid pAAV-miro-GFP-hGH-amp vector with CMV promoter (Applied Biological Materials, Richmond, BC, USA). The AAV8 viruses were packaged and tittered by Applied Biological Materials.

RNA isolation and gene expression quantification

Total RNA was extracted using TRIzol Reagent (Thermo Fisher Scientific), and cDNA was generated using a High-Capacity RNA-to-cDNA Kit (Applied Biosystems, Carlsbad, CA, USA).[19,20] qRT-PCR was performed on a QuantStudio 6 Fast real-time PCR system using Power SYBR Green PCR master mix (Applied Biosystems). Primers were designed using Primer3 Input software version 0.4.0. Primer sequences are listed in Table S1.

RNA sequencing and bioinformatics data analysis

RNA samples used for RNA sequencing were isolated from RAS/AKT-induced HCCs treated with AAV control or miR-22 as well as normal healthy livers from the same genetic background, FVB/N mice (n = 3). RNA was quantified with Nanodrop, and the quality was determined using a Qubit and Agilent RNA 6000 Nano Kit (Agilent Technologies, Santa Clara, CA, USA). Library preparation and sequencing were performed by Novogene (Sacramento, CA, USA). Libraries were prepared using a NEBNext Ultra II non-directional RNA Library Prep kit (New England Biolabs, Ipswich, MA, USA). Library quality and concentration were assessed with LabChip GX Touch nucleic acid analyzer (PerkinElmer, Waltham, MA, USA) and qPCR. Libraries were sequenced on Novaseq6000 using PE150 sequencing. Reads quality was checked using the fastqc (v0.11.7, https://www.bioinformatics.babraham.ac.uk/projects/fastqc/).[61]

RNA sequencing data was analyzed using the Salmon-tximport-DESeq2 pipeline. The pair-ended reads (FASTQ format) were mapped to the reference mouse genome assembly (GRCm39, GENCODE release 25) and quantified with Salmon.[62] Gene-level counts were imported with *tximport*,[63] and differential expression analysis was performed with DESeq2 (version 1.18) with the corrected p value <0.05 and fold change >1.5.[64] Pathway analysis was performed with iDEP (http://bioinformatics.sdstate.edu/idep93/) using GSEA method,[65,66] which is conducted in the pre-ranked mode using a recent faster algorithm based on the fgsea package (bioRxiv, http://biorxiv.org/content/early/2016/06/20/060012).[67] Functional pathways or processes with FDR <0.1 and Bonferroni value < 0.1 were accepted. KEGG gene set was used in the GSEA analysis.

TCGA analysis

miR-22 levels and survival analysis were performed using UCSC Xena (http://xena.ucsc.edu/) based on the TCGA LIHC dataset.[68] GSEA analysis based on the KEGG and hallmark gene set was performed by UCSC Xena differential gene expression analysis (http://analysis.xenahubs.net/).[68] The TCGA LIHC dataset includes 379 HCC and 59 adjacent normal liver specimens. For pathway analysis based on differentially expressed miR-22 levels, 181 HCC specimens were classified into two groups based on the upper and lower quartiles of miR-22 levels: (1) high miR-22 expression (miR-22 Hi) with $Log2^{(RPM)} > 17.48$ (n = 89) and (2) low miR-22 expression (miR-22 Lo) with $Log2^{(RPM)} < 16.5$ (n = 92).

Co-culture of hepatic T cells and HCC cells

Murine Hepa1-6 cells (ATCC CRL-1830) were seeded into 6-well plates at a density of 1 x 10^5/well in RPMI 1640 culture medium (Gibco, Grand Island, NY, USA) supplemented with 10% fetal bovine serum (R&D Systems, Minneapolis, MN, USA). At day 2, T cells isolated from livers of healthy, HCC, and miR-22-treated HCC mice were mixed with Hepa1-6 cells at a 1:1 ratio. Cells and supernatants were collected after 36 h of incubation for further flow cytometry or enzyme-linked immunosorbent assay (ELISA). For flow cytometry, 2 x 10^6 cells were stained with anti-CD3e antibody (BD Bioscience San Jose, California USA) to gate the $CD3^+$ T cell population. Annexin V/7-AAD staining (Thermo Fisher Scientific) was applied to determine the apoptosis of Hepa1-6 cells ($CD3^-$ cells) in round-bottom 96-well plates.

ELISA

The levels of IL17A, IL6, IFNγ, and granzyme B in the supernatant of the co-culture system were quantified by ELISA kits according to the manufacturer's protocol (Thermo Fisher Scientific).

Figure 5. Overexpression of IL23/IL17 attenuates the anti-HCC effect of miR-22

(A) Study design for miR-22 and IL23 overexpression in male HCC mice. (B) Representative liver morphology, (C) liver weight and L/B ratio, and (D) H&E-stained liver sections in each group (n = 8); scale bar, 100 μm. (E) mRNA levels of IL17A signaling-related genes in hepatic T cells isolated from indicated groups (n = 3). (F) Hepatic mRNA levels of HCC markers in each group (n = 3). Data represent mean ± SD. *p < 0.05, **p < 0.01, ***p < 0.001 by one-way ANOVA (C, E, and F).

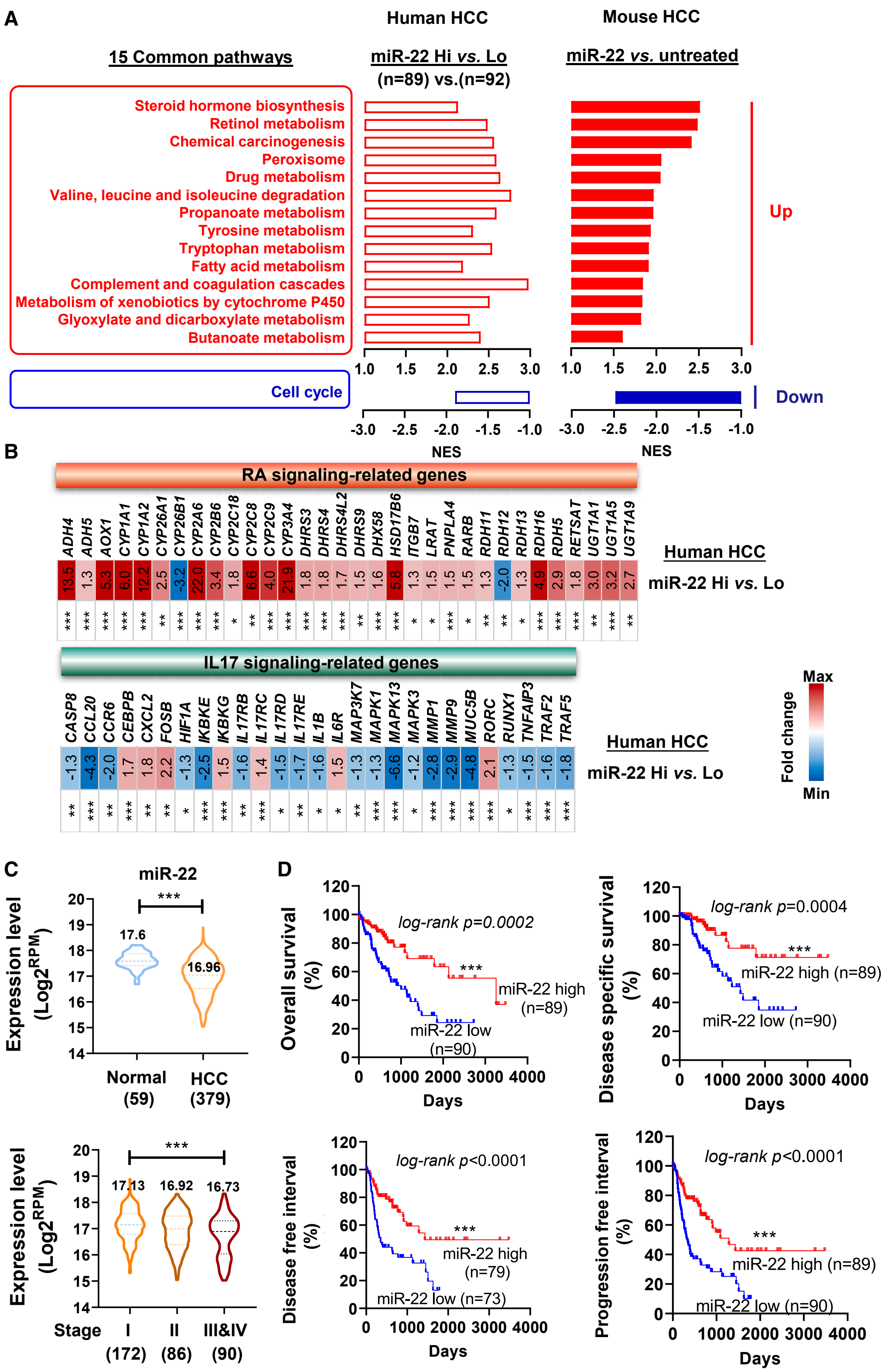

(legend on next page)

Flow cytometry

Antibodies used for flow cytometric analysis are listed in Table S1. They were tested for optimal dilution in-house based on lot, clone, and vendor. 2 x 10^6 lymphocytes isolated from the livers and spleens were stimulated with phorbol 12-myristate 13-acetate (PMA)/ionomycin (50 ng/mL and 500 ng/mL, respectively) in the presence of Golgi Stop (Thermo Fisher Scientific) for 6 h. Thereafter, the cells were washed and stained for surface antigens. Zombie Aqua fixable viability (1:500; BioLegend) was added to exclude dead cells. For surface antigen staining only, cells were fixed in 4% paraformaldehyde. For intracellular staining, cells were fixed and permeabilized using Foxp3/Transcription Factor Staining Buffer Set (eBioscience) followed by staining of intracellular proteins. Data were acquired by a BD LSRFortessa instrument running FACS DIVA software and analyzed with FlowJo v10.7.1 software (Tree Star, OR).

Chromatin immunoprecipitation-qPCR

ChIP-qPCR was performed based on a previous publication.[69] Briefly, chromatin lysate was precleared before incubation with anti-HIF1α (Novus Biologicals, Centennial, CO, USA), anti-RORγT (eBioscience), or anti-phosphor (P)-STAT3 (Cell Signaling Technology, Beverly, MA, USA). Rabbit IgG and H3Ac antibodies (MilliporeSigma, Burlington, MA, USA) were used as negative and positive controls, respectively. Samples were incubated with Dynase beads (Thermo Fisher Scientific) at 4°C overnight followed by de-crosslinking and purification. ChIP analysis was carried out according to the manufacturer's protocol (Upstate/Millipore, Massachusetts, USA). The immunoprecipitated DNA was quantified via RT-PCR with a QuantStudio 6 Fast real-time PCR system (Applied Biosystems) using SYBR Green. Primers and antibodies used for ChIP assays are listed in Tables S1 and S2.

Histology, tumor grade, Ki67 immunohistochemistry, and TUNEL assay

Tumor score was quantitively evaluated by pathologists based on H&E staining using five criteria including the level of centrilobular vacuolar degeneration, the number of proliferation foci, mitotic index, scirrhous type foci of proliferation, and inflammatory cells.[70,71] The tumor scoring criteria are described in Table S3.

Immunohistochemistry was performed as described previously.[28,72] To monitor hepatocyte proliferation, immunostaining was performed with anti-Ki67 antibody (NeoMarkers, Fremont, CA, USA) in healthy livers, HCC, miR-22-treated HCC, and lenvatinib-treated HCC. The number of proliferating hepatocytes was determined by counting positive-staining cells in at least five random microscopic fields (x10) for each specimen. Positive cells were determined using QuPath software.[73] To monitor apoptosis in response to miR-22, TUNEL assay was performed with TUNEL assay kit (Abcam, Cambridge, MA), according to the manufacturer's instructions.

Serum biochemistry analysis

Blood samples were collected at the endpoint of the experiments, and serum was separated within 2 h of the collection after centrifugation at 3,000 x *g* for 10 min. Serum ALT, AST, cholesterol, glucose, globulin, albumin, total protein, and BUN levels were quantified using FUJI DRI-CHEM 4000 veterinary chemistry analyzer (Heska Corporation, Loveland, CO) according to manufacturer's instruction.

Blood hematology analysis

Blood samples were collected at the endpoint of the experiments. 100 μL aliquots were analyzed within 10 min of collection using a veterinary Hematrue hematology analyzer (Heska Corporation) based on the manufacturer's instruction.[74]

Western blotting

Western blots were performed as described previously.[19,20,28] Proteins were extracted from the livers using a lysis buffer with cocktail protease inhibitors and phosphatase inhibitors (Thermo Fisher Scientific). Protein concentration was measured using the Pierce BCA protein assay kit, and 20–40 μg of total lysate was loaded and immunoblotted. Antibodies used were anti-IL6R (Santa Cruz Biotechnology, Santa Cruz, CA, USA), IL17A (eBioscience), phosphor (P)-STAT3, total (T)-STAT3 (Cell Signaling Technology), β-ACTIN (MilliporeSigma), and HIF1α (Novus Biologicals), which are listed in Table S1.

Cytotoxic CD8$^+$ T cell depletion assay

At 1 week post oncogene injection, HCC-bearing mice were randomly assigned into AAV-miR-22 or AAV blank treated groups. On the same day, HCC mice from each group were randomly divided into two groups to receive 200 μg of InVivoMAb anti-mouse CD8α (BE004-1, Bio X cell, Lebanon, NH, USA) or InVivoMAb rat IgG2a isotype control (BE0089, Bio X cell) via intraperitoneal injection twice per week for six times as indicated.[75]

IL-23 minicircle DNA production and hydrodynamic injection in HCC mice

Minicircle (MC)-RSV.Flag.mIL23.elasti.bpA or RSV.eGFP.bpA was produced to induce IL17 signaling in HCC mice as described.[76] Hydrodynamic delivery of 3 μg IL-23 or GFP MC DNA via tail vein injection was performed as previously described.[77] At 1 week post oncogene injection, HCC mice were randomly assigned into AAV-miR-22 or AAV blank treated groups. At 2 weeks post oncogene injection,

Figure 6. Mouse and human HCC have similar gene expression profiles based on miR-22 levels revealing human relevance of the findings
(A) Fifteen common pathways were identified by comparing miR-22 Hi (high, n = 89) vs. miR-22 Lo (low, n = 92) human HCC and miR-22 treated vs. untreated mouse HCC. (B) The fold changes of RA and IL17 signaling-related genes in miR-22 Hi vs. miR-22 Lo human HCC. (C) The levels of miR-22 in human HCC vs. normal livers and different stages of HCC. The numbers in parentheses are case numbers. Data were shown with medium ±5 to 95 percentiles. (D) Kaplan-Meier survival curves of HCC patients with high and low miR-22 levels based on TCGA LIHC. p values were calculated by the log rank test. *$p < 0.05$, **$p < 0.01$, ***$p < 0.001$ by unpaired two-tailed Student's t test (B and C).

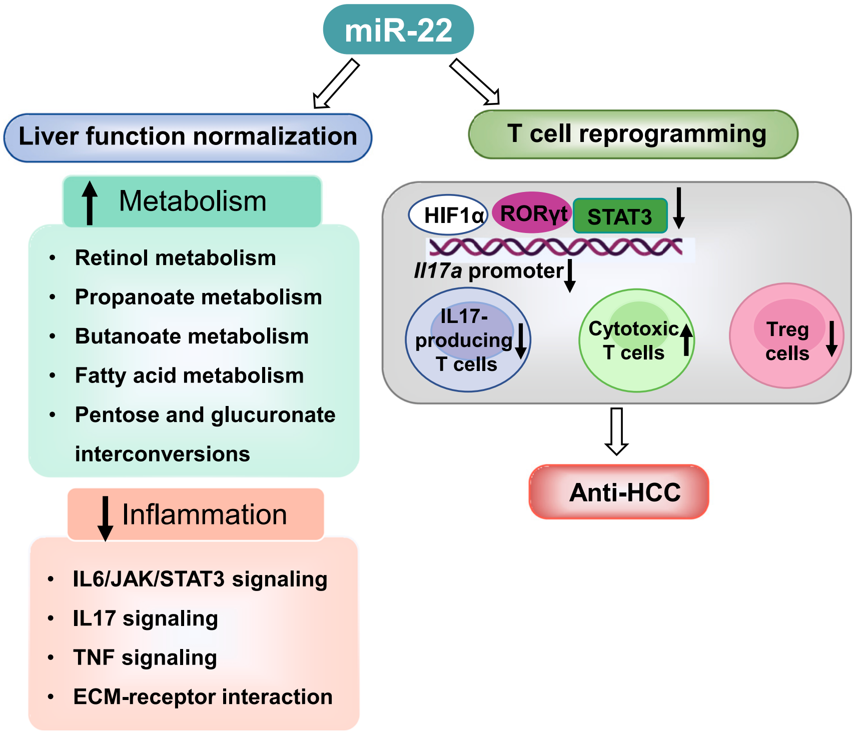

Figure 7. The schematic diagram summarizes miR-22 treats HCC by inducing metabolism and modulating T cell reprogramming

miR-22 treatment induces the metabolism of retinol, propanoate, butanoate, fatty acid, and sugar. Therefore, the miR-22 inducer signaling is compromised in HCC and restored due to positive treatment outcomes. Meanwhile, miR-22 inhibits inflammation pathways including IL17 signaling, cytokine-receptor interaction, and ECM-receptor interaction, which are all upregulated due to HCC development. In the T cells, miR-22 inhibits IL17 signaling at multiple levels: (1) miR-22 silences HIF1α and reduces its occupancy in the *Rorc* promoter. (2) miR-22 reduces *Rorc* expressions and the recruitment of RORγT/HIF1α to the *Il17a* gene leading to reduced expression. (3) miR-22 deactivates STAT3 and decreases its occupancy in the *Il17a* promoter, which consequentially reduces *Il17a* gene expression. Additionally, miR-22 reduces Treg cells. The reduced inflammatory signaling as well as immunosuppressive effects permit activation of cytotoxic T cells leading to cancer cell death.

mice from each subgroup were further divided into two groups to receive one dose (3 μg) of either IL-23 MC or MC control.

Statistical analysis

Statistical analysis was performed using Prism software v8.2.1 (Graph Software). Data were expressed as means ± standard deviation (SD). Statistical significance between two groups was evaluated using a two-tailed Student's t test. One-way ANOVA followed by Tukey's t test was used to compare the statistical difference among multiple groups. Associations were analyzed by linear regression. A value of $p < 0.05$ was considered statistically significant.

DATA AVAILABILITY

The source data that support the findings of this study are available. The RNA sequencing data have been deposited at the Gene Expression Omnibus under the accession number GSE215753. All the other data are available in the main text or supplemental information.

SUPPLEMENTAL INFORMATION

Supplemental information can be found online at https://doi.org/10.1016/j.ymthe.2023.04.019.

ACKNOWLEDGMENTS

We thank Drs. Snigdha Guha, Chengfei Liu, and Ms. Jennifer Clark from UC Davis for editing and commenting on the manuscript. We also thank Dr. Daisuke Yamada for preparing IL23 minicircle plasmids. We would like to thank the following funding sources for their support: National Institutes of Health, United States (R01CA222490, R50CA243787, and T32 CA108459-15).

AUTHOR CONTRIBUTIONS

Conceptualization, methodology, and study design: Y.-J.Y.W. and Y.H. Data acquisition, data analysis, and interpretation: Y.H., T.S., F.V., and Y.-J.Y.W. Manuscript writing: Y.H. and Y.-J.Y.W. Manuscript editing and commenting: T.S., X.C., X.S.W., and S.T.H. Obtaining research funding: Y.-J.Y.W. and Y.H.

DECLARATION OF INTERESTS

The authors declare no potential conflict of interest.

REFERENCES

1. Chen, B., Garmire, L., Calvisi, D.F., Chua, M.S., Kelley, R.K., and Chen, X. (2020). Harnessing big 'omics' data and AI for drug discovery in hepatocellular carcinoma. Nat. Rev. Gastroenterol. Hepatol. *17*, 238–251. https://doi.org/10.1038/s41575-019-0240-9.

2. Asrani, S.K., Devarbhavi, H., Eaton, J., and Kamath, P.S. (2019). Burden of liver diseases in the world. J. Hepatol. *70*, 151–171. https://doi.org/10.1016/j.jhep.2018.09.014.

3. Colquhoun, S.D., and Wan, Y.J.Y. (2020). Hepatocellular carcinoma diagnosis and treatment: an overview. Liver Res. *4*, 159–160. https://doi.org/10.1016/j.livres.2020.11.006.

4. Faivre, S., Rimassa, L., and Finn, R.S. (2020). Molecular therapies for HCC: looking outside the box. J. Hepatol. *72*, 342–352. https://doi.org/10.1016/j.jhep.2019.09.010.

5. Llovet, J.M., Kelley, R.K., Villanueva, A., Singal, A.G., Pikarsky, E., Roayaie, S., Lencioni, R., Koike, K., Zucman-Rossi, J., and Finn, R.S. (2021). Hepatocellular carcinoma. Nat. Rev. Dis. Primers *7*, 6. https://doi.org/10.1038/s41572-020-00240-3.

6. Xu, C., Xu, Z., Zhang, Y., Evert, M., Calvisi, D.F., and Chen, X. (2022). beta-Catenin signaling in hepatocellular carcinoma. J. Clin. Invest. *132*, e154515. https://doi.org/10.1172/JCI154515.

7. Nishida, N., and Kudo, M. (2018). Immune checkpoint blockade for the treatment of human hepatocellular carcinoma. Hepatol. Res. *48*, 622–634. https://doi.org/10.1111/hepr.13191.

8. Vaziri, F., Colquhoun, S., and Wan, Y.J.Y. (2020). Hepatocellular carcinoma immunotherapy: the impact of epigenetic drugs and the gut microbiome. Liver Res. *4*, 191–198. https://doi.org/10.1016/j.livres.2020.10.001.

9. Wang, J., Li, Y., Ding, M., Zhang, H., Xu, X., and Tang, J. (2017). Molecular mechanisms and clinical applications of miR-22 in regulating malignant progression in human cancer (Review). Int. J. Oncol. *50*, 345–355. https://doi.org/10.3892/ijo.2016.3811.

10. Mansini, A.P., Lorenzo Pisarello, M.J., Thelen, K.M., Cruz-Reyes, M., Peixoto, E., Jin, S., Howard, B.N., Trussoni, C.E., Gajdos, G.B., LaRusso, N.F., et al. (2018). MicroRNA (miR)-433 and miR-22 dysregulations induce histone-deacetylase-6 overexpression and ciliary loss in cholangiocarcinoma. Hepatology *68*, 561–573. https://doi.org/10.1002/hep.29832.

11. Liu, Y., Chen, X., Cheng, R., Yang, F., Yu, M., Wang, C., Cui, S., Hong, Y., Liang, H., Liu, M., et al. (2018). The Jun/miR-22/HuR regulatory axis contributes to tumourigenesis in colorectal cancer. Mol. Cancer *17*, 11. https://doi.org/10.1186/s12943-017-0751-3.

12. Xia, S., Wang, X., Wu, Y., Zhou, T., Tian, H., Liu, Z., Li, L., Yan, Z., and Zhang, G. (2022). miR-22 suppresses EMT by mediating metabolic reprogramming in colorectal cancer through targeting MYC-associated factor X. Dis. Markers *2022*, 7843565. https://doi.org/10.1155/2022/7843565.

13. Yang, X., Su, W., Li, Y., Zhou, Z., Zhou, Y., Shan, H., Han, X., Zhang, M., Zhang, Q., Bai, Y., et al. (2021). MiR-22-3p suppresses cell growth via MET/STAT3 signaling in lung cancer. Am. J. Transl. Res. *13*, 1221–1232.

14. Konishi, H., Hayashi, M., Taniguchi, K., Nakamura, M., Kuranaga, Y., Ito, Y., Kondo, Y., Sasaki, H., Terai, Y., Akao, Y., and Ohmichi, M. (2020). The therapeutic potential of exosomal miR-22 for cervical cancer radiotherapy. Cancer Biol. Ther. *21*, 1128–1135. https://doi.org/10.1080/15384047.2020.1838031.

15. Zhang, W., Zhan, F., Li, D., Wang, T., and Huang, H. (2020). RGMB-AS1/miR-22-3p/NFIB axis contributes to the progression of gastric cancer. Neoplasma *67*, 484–491. https://doi.org/10.4149/neo_2020_190418N350.

16. Vesuna, F., Lisok, A., van Diest, P., and Raman, V. (2021). Twist activates miR-22 to suppress estrogen receptor alpha in breast cancer. Mol. Cell. Biochem. *476*, 2295–2306. https://doi.org/10.1007/s11010-021-04065-w.

17. Gao, Y., Li, X., Zeng, C., Liu, C., Hao, Q., Li, W., Zhang, K., Zhang, W., Wang, S., Zhao, H., et al. (2020). CD63(+) cancer-associated fibroblasts confer tamoxifen resistance to breast cancer cells through exosomal miR-22. Adv. Sci. *7*, 2002518. https://doi.org/10.1002/advs.202002518.

18. Jiang, R., Deng, L., Zhao, L., Li, X., Zhang, F., Xia, Y., Gao, Y., Wang, X., and Sun, B. (2011). miR-22 promotes HBV-related hepatocellular carcinoma development in males. Clin. Cancer Res. *17*, 5593–5603. https://doi.org/10.1158/1078-0432.CCR-10-1734.

19. Yang, F., Hu, Y., Liu, H.X., and Wan, Y.J.Y. (2015). MiR-22-silenced cyclin A expression in colon and liver cancer cells is regulated by bile acid receptor. J. Biol. Chem. *290*, 6507–6515. https://doi.org/10.1074/jbc.M114.620369.

20. Hu, Y., French, S.W., Chau, T., Liu, H.X., Sheng, L., Wei, F., Stondell, J., Garcia, J.C., Du, Y., Bowlus, C.L., and Wan, Y.J.Y. (2019). RARbeta acts as both an upstream regulator and downstream effector of miR-22, which epigenetically regulates NUR77 to induce apoptosis of colon cancer cells. FASEB J. *33*, 2314–2326. https://doi.org/10.1096/fj.201801390R.

21. Alvarez-Díaz, S., Valle, N., Ferrer-Mayorga, G., Lombardía, L., Herrera, M., Domínguez, O., Segura, M.F., Bonilla, F., Hernando, E., and Muñoz, A. (2012). MicroRNA-22 is induced by vitamin D and contributes to its antiproliferative, antimigratory and gene regulatory effects in colon cancer cells. Hum. Mol. Genet. *21*, 2157–2165. https://doi.org/10.1093/hmg/dds031.

22. Sheng, L., Jena, P.K., Hu, Y., Liu, H.X., Nagar, N., Kalanetra, K.M., French, S.W., French, S.W., Mills, D.A., and Wan, Y.J.Y. (2017). Hepatic inflammation caused by dysregulated bile acid synthesis is reversible by butyrate supplementation. J. Pathol. *243*, 431–441. https://doi.org/10.1002/path.4983.

23. Liu, H.X., Keane, R., Sheng, L., and Wan, Y.J.Y. (2015). Implications of microbiota and bile acid in liver injury and regeneration. J. Hepatol. *63*, 1502–1510. https://doi.org/10.1016/j.jhep.2015.08.001.

24. Wan, Y.J.Y., and Jena, P.K. (2019). Precision dietary supplementation based on personal gut microbiota. Nat. Rev. Gastroenterol. Hepatol. *16*, 204–206. https://doi.org/10.1038/s41575-019-0108-z.

25. Zhan, L., Liu, H.X., Fang, Y., Kong, B., He, Y., Zhong, X.B., Fang, J., Wan, Y.J.Y., and Guo, G.L. (2014). Genome-wide binding and transcriptome analysis of human farnesoid X receptor in primary human hepatocytes. PLoS One *9*, e105930. https://doi.org/10.1371/journal.pone.0105930.

26. Gyamfi, M.A., He, L., French, S.W., Damjanov, I., and Wan, Y.J.Y. (2008). Hepatocyte retinoid X receptor alpha-dependent regulation of lipid homeostasis and inflammatory cytokine expression contributes to alcohol-induced liver injury. J. Pharmacol. Exp. Ther. *324*, 443–453. https://doi.org/10.1124/jpet.107.132258.

27. Luo, L.J., Zhang, L.P., Duan, C.Y., Wang, B., He, N.N., Abulimiti, P., and Lin, Y. (2017). The inhibition role of miR-22 in hepatocellular carcinoma cell migration and invasion via targeting CD147. Cancer Cel Int. *17*, 17. https://doi.org/10.1186/s12935-016-0380-8.

28. Hu, Y., Liu, H.X., Jena, P.K., Sheng, L., Ali, M.R., and Wan, Y.J.Y. (2020). miR-22 inhibition reduces hepatic steatosis via FGF21 and FGFR1 induction. JHEP Rep. *2*, 100093. https://doi.org/10.1016/j.jhepr.2020.100093.

29. Wang, L., Wang, Y.S., Mugiyanto, E., Chang, W.C., and Yvonne Wan, Y.J. (2020). MiR-22 as a metabolic silencer and liver tumor suppressor. Liver Res. *4*, 74–80. https://doi.org/10.1016/j.livres.2020.06.001.

30. Tian, J., Wang, W., Zhu, J., Zhuang, Y., Qi, C., Cai, Z., Yan, W., Lu, W., and Shang, A. (2022). Histone methyltransferase SETDB1 promotes immune evasion in colorectal cancer via FOSB-mediated downregulation of MicroRNA-22 through BATF3/PD-L1 pathway. J. Immunol. Res. *2022*, 4012920. https://doi.org/10.1155/2022/4012920.

31. Ma, X., Zhou, J., Zhong, Y., Jiang, L., Mu, P., Li, Y., Singh, N., Nagarkatti, M., and Nagarkatti, P. (2014). Expression, regulation and function of microRNAs in multiple sclerosis. Int. J. Med. Sci. *11*, 810–818. https://doi.org/10.7150/ijms.8647.

32. Lu, W., You, R., Yuan, X., Yang, T., Samuel, E.L.G., Marcano, D.C., Sikkema, W.K.A., Tour, J.M., Rodriguez, A., Kheradmand, F., and Corry, D.B. (2015). The microRNA miR-22 inhibits the histone deacetylase HDAC4 to promote T(H)17 cell-dependent emphysema. Nat. Immunol. *16*, 1185–1194. https://doi.org/10.1038/ni.3292.

33. Stauffer, J.K., Scarzello, A.J., Andersen, J.B., De Kluyver, R.L., Back, T.C., Weiss, J.M., Thorgeirsson, S.S., and Wiltrout, R.H. (2011). Coactivation of AKT and beta-catenin in mice rapidly induces formation of lipogenic liver tumors. Cancer Res. *71*, 2718–2727. https://doi.org/10.1158/0008-5472.CAN-10-2705.

34. Chai, Z.T., Zhang, X.P., Shao, M., Ao, J.Y., Chen, Z.H., Zhang, F., Hu, Y.R., Zhong, C.Q., Lin, J.H., Fang, K.P., et al. (2021). Impact of splenomegaly and splenectomy on prognosis in hepatocellular carcinoma with portal vein tumor thrombus treated with hepatectomy. Ann. Transl. Med. *9*, 247. https://doi.org/10.21037/atm-20-2229.

35. Xu, L., Yang, W., Shu, Y.F., and Xu, X.F. (2020). Hepatocellular carcinoma and multiple myeloma with elevated globulin: a case report and literature review. J. Int. Med. Res. *48*, 300060520920395. https://doi.org/10.1177/0300060520920395.

36. Regino, C.A., López-Montoya, V., López-Urbano, F., Alvarez, J.C., and Roman-Gonzalez, A. (2020). Paraneoplastic hypoglycemia in hepatocarcinoma: case report and literature review. Cureus *12*, e12013. https://doi.org/10.7759/cureus.12013.

37. Yang, J., Yan, B., Yang, L., Li, H., Fan, Y., Zhu, F., Zheng, J., and Ma, X. (2018). Macrocytic anemia is associated with the severity of liver impairment in patients with hepatitis B virus-related decompensated cirrhosis: a retrospective cross-sectional study. BMC Gastroenterol. *18*, 161. https://doi.org/10.1186/s12876-018-0893-9.

38. Ruiz de Galarreta, M., Bresnahan, E., Molina-Sánchez, P., Lindblad, K.E., Maier, B., Sia, D., Puigvehi, M., Miguela, V., Casanova-Acebes, M., Dhainaut, M., et al. (2019). Beta-catenin activation promotes immune escape and resistance to anti-PD-1 therapy in hepatocellular carcinoma. Cancer Discov. *9*, 1124–1141. https://doi.org/10.1158/2159-8290.CD-19-0074.

39. Wilson, G.K., Tennant, D.A., and McKeating, J.A. (2014). Hypoxia inducible factors in liver disease and hepatocellular carcinoma: current understanding and future directions. J. Hepatol. *61*, 1397–1406. https://doi.org/10.1016/j.jhep.2014.08.025.

40. Chen, C., and Lou, T. (2017). Hypoxia inducible factors in hepatocellular carcinoma. Oncotarget *8*, 46691–46703. https://doi.org/10.18632/oncotarget.17358.

41. Yamakuchi, M., Yagi, S., Ito, T., and Lowenstein, C.J. (2011). MicroRNA-22 regulates hypoxia signaling in colon cancer cells. PLoS One *6*, e20291. https://doi.org/10.1371/journal.pone.0020291.

42. Bao, M.H., and Wong, C.C. (2021). Hypoxia, metabolic reprogramming, and drug resistance in liver cancer. Cells *10*, e71715. https://doi.org/10.3390/cells10071715.

43. Narravula, S., and Colgan, S.P. (2001). Hypoxia-inducible factor 1-mediated inhibition of peroxisome proliferator-activated receptor alpha expression during hypoxia. J. Immunol. *166*, 7543–7548. https://doi.org/10.4049/jimmunol.166.12.7543.

44. Bansal, A., Harris, R.A., and DeGrado, T.R. (2012). Choline phosphorylation and regulation of transcription of choline kinase alpha in hypoxia. J. Lipid Res. *53*, 149–157. https://doi.org/10.1194/jlr.M021030.

45. Lequeux, A., Noman, M.Z., Xiao, M., Van Moer, K., Hasmim, M., Benoit, A., Bosseler, M., Viry, E., Arakelian, T., Berchem, G., et al. (2021). Targeting HIF-1 alpha transcriptional activity drives cytotoxic immune effector cells into melanoma and improves combination immunotherapy. Oncogene *40*, 4725–4735. https://doi.org/10.1038/s41388-021-01846-x.

46. Kansy, B.A., Concha-Benavente, F., Srivastava, R.M., Jie, H.B., Shayan, G., Lei, Y., Moskovitz, J., Moy, J., Li, J., Brandau, S., et al. (2017). PD-1 status in CD8(+) T cells associates with survival and anti-PD-1 therapeutic outcomes in head and neck cancer. Cancer Res. *77*, 6353–6364. https://doi.org/10.1158/0008-5472.CAN-16-3167.

47. Xiao, S., Jin, H., Korn, T., Liu, S.M., Oukka, M., Lim, B., and Kuchroo, V.K. (2008). Retinoic acid increases Foxp3+ regulatory T cells and inhibits development of Th17 cells by enhancing TGF-beta-driven Smad3 signaling and inhibiting IL-6 and IL-23 receptor expression. J. Immunol. *181*, 2277–2284. https://doi.org/10.4049/jimmunol.181.4.2277.

48. Hammerschmidt, S.I., Friedrichsen, M., Boelter, J., Lyszkiewicz, M., Kremmer, E., Pabst, O., and Förster, R. (2011). Retinoic acid induces homing of protective T and B cells to the gut after subcutaneous immunization in mice. J. Clin. Invest. *121*, 3051–3061. https://doi.org/10.1172/JCI44262.

49. Suthar, M.S., Ramos, H.J., Brassil, M.M., Netland, J., Chappell, C.P., Blahnik, G., McMillan, A., Diamond, M.S., Clark, E.A., Bevan, M.J., and Gale, M., Jr. (2012). The RIG-I-like receptor LGP2 controls CD8(+) T cell survival and fitness. Immunity *37*, 235–248. https://doi.org/10.1016/j.immuni.2012.07.004.

50. Wang, D., Yu, W., Lian, J., Wu, Q., Liu, S., Yang, L., Li, F., Huang, L., Chen, X., Zhang, Z., et al. (2020). Th17 cells inhibit CD8(+) T cell migration by systematically downregulating CXCR3 expression via IL-17A/STAT3 in advanced-stage colorectal cancer patients. J. Hematol. Oncol. *13*, 68. https://doi.org/10.1186/s13045-020-00897-z.

51. Dang, E.V., Barbi, J., Yang, H.Y., Jinasena, D., Yu, H., Zheng, Y., Bordman, Z., Fu, J., Kim, Y., Yen, H.R., et al. (2011). Control of T(H)17/T(reg) balance by hypoxia-inducible factor 1. Cell *146*, 772–784. https://doi.org/10.1016/j.cell.2011.07.033.

52. Yang, X.P., Ghoreschi, K., Steward-Tharp, S.M., Rodriguez-Canales, J., Zhu, J., Grainger, J.R., Hirahara, K., Sun, H.W., Wei, L., Vahedi, G., et al. (2011). Opposing regulation of the locus encoding IL-17 through direct, reciprocal actions of STAT3 and STAT5. Nat. Immunol. *12*, 247–254. https://doi.org/10.1038/ni.1995.

53. Shi, Z., Wu, X., Wu, C.Y., Singh, S.P., Law, T., Yamada, D., Huynh, M., Liakos, W., Yang, G., Farber, J.M., et al. (2022). Bile acids improve psoriasiform dermatitis through inhibition of IL-17a expression and CCL20-CCR6-mediated trafficking of T cells. J. Invest. Dermatol. *142*, 1381–1390.e11. https://doi.org/10.1016/j.jid.2021.10.027.

54. Togashi, Y., Shitara, K., and Nishikawa, H. (2019). Regulatory T cells in cancer immunosuppression - implications for anticancer therapy. Nat. Rev. Clin. Oncol. *16*, 356–371. https://doi.org/10.1038/s41571-019-0175-7.

55. Bushue, N., and Wan, Y.J.Y. (2010). Retinoid pathway and cancer therapeutics. Adv. Drug Deliv. Rev. *62*, 1285–1298. https://doi.org/10.1016/j.addr.2010.07.003.

56. Brown, C.C., Esterhazy, D., Sarde, A., London, M., Pullabhatla, V., Osma-Garcia, I., Al-Bader, R., Ortiz, C., Elgueta, R., Arno, M., et al. (2015). Retinoic acid is essential for Th1 cell lineage stability and prevents transition to a Th17 cell program. Immunity *42*, 499–511. https://doi.org/10.1016/j.immuni.2015.02.003.

57. Wang, Y., Liu, T., Tang, W., Deng, B., Chen, Y., Zhu, J., and Shen, X. (2016). Hepatocellular carcinoma cells induce regulatory T cells and lead to poor prognosis via production of transforming growth factor-beta1. Cell. Physiol. Biochem. *38*, 306–318. https://doi.org/10.1159/000438631.

58. Unitt, E., Rushbrook, S.M., Marshall, A., Davies, S., Gibbs, P., Morris, L.S., Coleman, N., and Alexander, G.J.M. (2005). Compromised lymphocytes infiltrate hepatocellular carcinoma: the role of T-regulatory cells. Hepatology *41*, 722–730. https://doi.org/10.1002/hep.20644.

59. Chen, X., and Calvisi, D.F. (2014). Hydrodynamic transfection for generation of novel mouse models for liver cancer research. Am. J. Pathol. *184*, 912–923. https://doi.org/10.1016/j.ajpath.2013.12.002.

60. Wiede, F., and Tiganis, T. (2018). Isolation and characterization of mouse intrahepatic lymphocytes by flow cytometry. Methods Mol. Biol. *1725*, 301–311. https://doi.org/10.1007/978-1-4939-7568-6_23.

61. Aronesty, E. (2013). Comparison of sequencing utility programs. Open Bioinforma. J. 7, 1–8.

62. Patro, R., Duggal, G., Love, M.I., Irizarry, R.A., and Kingsford, C. (2017). Salmon provides fast and bias-aware quantification of transcript expression. Nat. Methods *14*, 417–419. https://doi.org/10.1038/nmeth.4197.

63. Soneson, C., Love, M.I., and Robinson, M.D. (2015). Differential analyses for RNA-seq: transcript-level estimates improve gene-level inferences. F1000Res. *4*, 1521. https://doi.org/10.12688/f1000research.7563.2.

64. Love, M.I., Huber, W., and Anders, S. (2014). Moderated estimation of fold change and dispersion for RNA-seq data with DESeq2. Genome Biol. *15*, 550. https://doi.org/10.1186/s13059-014-0550-8.

65. Subramanian, A., Tamayo, P., Mootha, V.K., Mukherjee, S., Ebert, B.L., Gillette, M.A., Paulovich, A., Pomeroy, S.L., Golub, T.R., Lander, E.S., and Mesirov, J.P. (2005). Gene set enrichment analysis: a knowledge-based approach for interpreting genome-wide expression profiles. Proc. Natl. Acad. Sci. USA *102*, 15545–15550. https://doi.org/10.1073/pnas.0506580102.

66. Ge, S.X., Son, E.W., and Yao, R. (2018). iDEP: an integrated web application for differential expression and pathway analysis of RNA-Seq data. BMC Bioinformatics *19*, 534. https://doi.org/10.1186/s12859-018-2486-6.

67. Sergushichev, A.A. (2016). An algorithm for fast preranked gene set enrichment analysis using cumulative statistic calculation. Preprint at bioRxiv. https://doi.org/10.1101/060012.

68. Goldman, M.J., Craft, B., Hastie, M., Repečka, K., McDade, F., Kamath, A., Banerjee, A., Luo, Y., Rogers, D., Brooks, A.N., et al. (2020). Visualizing and interpreting cancer genomics data via the Xena platform. Nat. Biotechnol. *38*, 675–678. https://doi.org/10.1038/s41587-020-0546-8.

69. Hu, Y., Liu, H.X., He, Y., Fang, Y., Fang, J., and Wan, Y.J.Y. (2013). Transcriptome profiling and genome-wide DNA binding define the differential role of fenretinide and all-trans RA in regulating the death and survival of human hepatocellular carcinoma Huh7 cells. Biochem. Pharmacol. *85*, 1007–1017. https://doi.org/10.1016/j.bcp.2013.01.023.

70. Thoolen, B., Maronpot, R.R., Harada, T., Nyska, A., Rousseaux, C., Nolte, T., Malarkey, D.E., Kaufmann, W., Küttler, K., Deschl, U., et al. (2010). Proliferative and nonproliferative lesions of the rat and mouse hepatobiliary system. Toxicol. Pathol. *38*, 5S–81S. https://doi.org/10.1177/0192623310386499.

71. Ha, S.Y., Choi, M., Lee, T., and Park, C.K. (2016). The prognostic role of mitotic index in hepatocellular carcinoma patients after curative hepatectomy. Cancer Res. Treat. *48*, 180–189. https://doi.org/10.4143/crt.2014.321.

72. Hu, Y., Zhan, Q., Liu, H.X., Chau, T., Li, Y., and Wan, Y.J. (2014). Accelerated partial hepatectomy-induced liver cell proliferation is associated with liver injury in Nur77 knockout mice. Am. J. Pathol. *184*, 3272–3283. https://doi.org/10.1016/j.ajpath.2014.08.002.

73. Bankhead, P., Loughrey, M.B., Fernández, J.A., Dombrowski, Y., McArt, D.G., Dunne, P.D., McQuaid, S., Gray, R.T., Murray, L.J., Coleman, H.G., et al. (2017). QuPath: open source software for digital pathology image analysis. Sci. Rep. *7*, 16878. https://doi.org/10.1038/s41598-017-17204-5.

74. Mohr, A.M., ElHassan, I.O., Hannoush, E.J., Sifri, Z.C., Offin, M.D., Alzate, W.D., Rameshwar, P., and Livingston, D.H. (2011). Does beta blockade postinjury prevent bone marrow suppression? J. Trauma *70*, 1043–1049. https://doi.org/10.1097/TA.0b013e3182169326.

75. Wen, L., Xin, B., Wu, P., Lin, C.H., Peng, C., Wang, G., Lee, J., Lu, L.F., and Feng, G.S. (2019). An efficient combination immunotherapy for primary liver cancer by harmonized activation of innate and adaptive immunity in mice. Hepatology *69*, 2518–2532. https://doi.org/10.1002/hep.30528.

76. Chen, Z.Y., He, C.Y., and Kay, M.A. (2005). Improved production and purification of minicircle DNA vector free of plasmid bacterial sequences and capable of persistent transgene expression in vivo. Hum. Gene Ther. *16*, 126–131. https://doi.org/10.1089/hum.2005.16.126.

77. Adamopoulos, I.E., Tessmer, M., Chao, C.C., Adda, S., Gorman, D., Petro, M., Chou, C.C., Pierce, R.H., Yao, W., Lane, N.E., et al. (2011). IL-23 is critical for induction of arthritis, osteoclast formation, and maintenance of bone mass. J. Immunol. *187*, 951–959. https://doi.org/10.4049/jimmunol.1003986.

YMTHE, Volume *31*

Supplemental Information

miR-22 gene therapy treats HCC
by promoting anti-tumor immunity
and enhancing metabolism

Ying Hu, Tahereh Setayesh, Farzam Vaziri, Xuesong Wu, Samuel T. Hwang, Xin Chen, and Yu-Jui Yvonne Wan

Table S1. Antibodies used for IHC, Western blot, ChIP, or flow cytometry.

Name	Citation	Supplier	Cat no.	Clone no.
IL6Rα	28	Santa Cruz Biotechnology	sc-660	
Phospho-Stat3 (Tyr705)	1236	Cell Signaling Technology	#9131	
Stat3 (124H6) Mouse mAb	1338	Cell Signaling Technology	#9139	124H6
IL17A	20	Invitrogen eBioscience	14-7175-81	eBio17CK15A5
HIF1α	862	Novus Biologicals	NB100-105	H1alpha67
β-ACTIN	3568	MilliporeSigma	A1978	
Ki-67	264	NeoMarkers	RB-1510-P	
CD45-PE/Cy7	144	Biolegend	103114	30-F11
CD3-BuV395	30	BD Biosciences	563565	145-2C11
CD4-BV605	17	BD Biosciences	563151	RM4-5
CD8-Alexa700	27	BD Biosciences	557959	53-6.7
CD44-BV711	9	BD Biosciences	563971	IM7
CD62L-Alexa647	8	Biolegend	104421	MEL-14
CD25-PE	290	Invitrogen eBioscience	12-0251-82	PC61.5
FoxP3-FITC	376	Invitrogen eBioscience	11-5773-82	FJK-16s
IL17A-PECF594	6	BD Biosciences	562542	TC11-18H10
CD107a-BV711	0	BD Biosciences	564348	1D4B
CD45-PE	125	Biolegend	103106	30-F11
CD3-PerCP Cy5.5	67	BD Biosciences	551163	145-2C11
CD8-FITC	32	Invitrogen	MA5-17597	CT-CD8a
CD4-APC	161	Biolegend	100412	GK1.5
InVivoMAb anti-mouse CD8α	80	Bio X Cell	BE0004-1	53-6.7
InVivoMAb rat IgG2a isotype control	367	Bio X Cell	BE0089	
IFNγ-PE	2	BD Biosciences	612769	XMG1.2
CD3-APC	132	BD Biosciences	553066	145-2C11
RORγ	129	Invitrogen eBioscience	12-6988-82	AFKJS-9
Normal Mouse IgG	329	MilliporeSigma	13-371	
Normal Rabbit IgG	641	MilliporeSigma	12-370	
RNA Polymerase II	195	MilliporeSigma	05-623	CTD4H8

Table S2. Primer sequences.

Primers	Sequence	
Primers used for qRT-PCR		
Afp	F 5'-CAGTGCGTGACGGAGAAGAA -3'	R 5'-AAACACCCATCGCCAGAGTT-3'
Cd133	F 5'-TGATTCCAAGGAGATTGCCCT-3'	R 5'-GCAGCAACGGCACATACAAA-3'
Ccna2	F 5'-ACAGAGCTGGCCTGAGTCAT-3'	R 5'-TTGACTGTTGGGCATGTTGT-3'
Gpc3	F 5'-AATCAACTGCGCTTCCTTGC-3'	R 5'-AGGTGGTGATCTCGTTGTCC-3'
Alb	F 5'-TACAGCGGAGCAACTGAAGA-3'	R 5'-TTGCAGCACAGAGACAAGAA-3'
Cyp3a11	F 5'-TCACAGACCCAGAGACGATTAAGA-3'	R 5'-CCCGCCGGTTTGTGAAG-3'
Cd3e	F 5'- TCTCGGAAGTCGAGGACAGT3'	R 5'-ATCAGCAAGCCCAGAGTGAT-3'
Cd4	F 5'- ACACACCTGTGCAAGAAGCA-3'	R 5'-GCTCTTGTTGGTTGGGAATC-3'
Cd8a	F 5'-CTCACCTGTGCACCCTACC-3'	R 5'-ATCCGGTCCCCTTCACTG-3'
Il17a	F 5'-TTTAACTCCCTTGGCGCAAAA-3'	R 5'-CTTTCCCTCCGCATTGACAC-3'
Il17f	F 5'-CTGGAGGATAACACTGTGAGAGT-3'	R 5'-TGCTGAATGGCGACGGAGTTC-3'
Rorc	F 5'-TCCCGAGATGCTGTCAAGTT-3'	R 5'-ACTTGTTCCTGTTGCTGCTG-3'
Ccl20	F 5'-ACTGTTGCCTCTCGTACATACA-3'	R 5'-ACCCACAATAGCTCTGGAAGG-3'
Ccr6	F 5'-GTCACTGTCATGCTTACTTGAATG-3'	R 5'-CTTAGGACTGGAGCCTGGATA-3'
Il6	F 5'-GTTGCCTTCTTGGGACTGATG-3'	R 5'-GGGAGTGGTATCCTCTGTGAAGTCT-3'
Il6ra	F 5'-ACAGTGTGGGAAGCAAGTCC-3'	R 5'-TCGGTATCGAAGCTGGAACT-3'
Il23a	F 5'-TGAAGATGTCAGAGTCAAGCAG-3'	R 5'-ACAAGGACTCAAGGACAACAG-3'
Il23r	F 5'-AAGGCTTTTCGGAACCTCAT-3'	R 5'-TTCCAGGTGCATGTCATGTT-3'
Il22	F 5'-TTGAGGTGTCCAACTTCCAGCA -3'	R 5'-AGCCGGACGTCTGTGTTGTTA-3'
S100a8	F 5'-TGTCCTCAGTTTGTGCAGAATATAAA-3'	R 5'-TCACCATCGCAAGGAACTCC-3'
S100A9	F 5'-GGTGGAAGCACAGTTGGCA-3'	R 5'-GTGTCCAGGTCCTCCATGATG-3'
Hif1a	F 5'-TCATCCATGTGACCATGAGG-3'	R 5'-AAAAAGCTCCGCTGTGTGTT-3'
Tgfb1	F 5'- GCCTGAGTGGCTGTCTTTTGACG-3'	R 5'-ACTTCCAACCCAGGTCCTTC-3'
Foxp3	F 5'-TCCTTCCCAGAGTTCTTCCA-3'	R 5'-CGAACATGCGAGTAAACCAA -3'
Il2ra	F 5'-AACGGCACCATCCTAAACTG-3'	R 5'-CTGTGTTGGCTTCTGCATGT-3'
Il2	F 5'-AGGAACCTGAAACTCCCCAG-3'	R 5'- AAATCCAGAACATGCCGCAG-3'
Nt5e	F 5'-AGGTTGTGGGGATTGTTGGA-3'	R 5'-CCCCAGGGCGATGATCTTAT-3'
Lag3	F 5'-CCTCGATGATTGCTAGTCCCT-3'	R 5'-GTAGACAGGCACTCGGTTCTG-3'
Nrp1	F 5'-AATGTTCTGTCGCTATGACCGGCT-3'	R 5'-TTCTGCCCACAATAACGCCCAATG-3'
Ctla4	F 5'-CATGTACCCACCGCCATACT-3'	R 5'-CCAAGCTAACTGCGACAAGG-3'
Il10	F 5'-GGAGCAGGTGAAGAGTGATTTTAATA-3'	R 5'-TGCAGTTGATGAAGATGTCAAATTC-3'
Il12a	F 5'-TGATGATGACCCTGTGCCTT-3'	R 5'-CGCAGAGTCTCGCCATTATG-3'
Il35b	F 5'-GATCCACGTCCTTCATTGCC-3'	R 5'-TGATTCGCTCAGCCACAAAG-3'
Runx1	F 5'-AGCGAGATTCAACGACCTCA-3'	R 5'-GCCGTCCACTGTGATTTTGA-3'
Itga4	F 5'-GCCTGGAGGAGAGGGATAAC-3'	R 5'-CAGAAGGCATGACGTAGCAA-3'
Itgb7	F 5'-CTACGACTCTGGGCTCTTGG-3'	R 5'-ACAGGTCAGCCTCAGAGCAT-3'
CCr9	F 5'-CTTGCCACTCTTCCCTTCTG-3'	R 5'-GCCTTCATGGCCTGTACAAT-3'
Ccl25	F 5'-CCAAGGTGCCTTTGAAGACT-3'	R 5'-TCCTCCAGCTGGTGGTTACT-3'
Madcam1	F 5'-GCATGGTGACCTGGCAGTGAAG-3'	R 5'-GGCAGCAGTATCCTCTCTGTAC-3'

Ifih1	F 5’-GGAAACAGCGGGAATGAGTC-3’	R 5’-AGCAGGCAGAAGACACTCAT-3’
Dhx58	F 5’-GTAGACAGAGGCAAGGTGGT-3’	R 5’-TACAGATGAGCAGGTCGTGG-3’
Rarb	F 5’-GCACTGACGCCATAGTGGTA-3’	R 5’-CACCATCTCCACTTCCTCCT-3’
Cyp26a1	F 5’-GCACAAGCAGCGAAAGAAGGTGAT-3’	R 5’-ACTGCTCCAGACAACTGCTGACTT-3’
Cyp26b1	F 5’-CGGAGAGACTGGTCACTGGT-3’	R 5’-CGCCCCAGTAAGTGTGTCTT-3’
Gapdh	F 5’-TGTGTCCGTCGTGGATCTGA-3’	R 5’-CCTGCTTCACCACCTTCTTGA-3’
18s	F 5’-CCGAAGCGTTTTACTTTGAAAAAA-3’	R 5’-TTCATTATTCCTAGCTGCGGTATC-3’
miR-22	F 5’-CGCGAAGCTGCCAGTTGAAG-3’	R 5’-GTGCAGGGTCCGAGGT-3’
U6	F 5’-CTCGCTTCGGCAGCACA-3’	R 5’-AACGCTTCACGAATTTGCGT-3’

Primers used for ChIP-qPCR	
RORγt _P1 (-4149 to -4208) HIF1α- ChIP	F 5’-CACCTCCAGGTTGTTTGCCCC-3’
	R 5’-GAGTGTGCATGTCTGTGGAGG-3’
RORγt_P2 (-6988 to -7002) HIF1α- ChIP	F 5’-GCGAAGGGACAGCTGCCTGC-3’
	R 5’-CTCCAGCTGGTAAACAGCAG-3’
RORγt _P3 (non-HIF1 binding) HIF1α- ChIP	F 5’-CAATCCTCCGTGCTGACAGCA-3’
	R 5’-CTGTCTAAGGGCGAAGGTCA-3’
IL17A _P1 (-4739 to -4755) p-STAT3-ChIP	F 5’- CAGGTATTATTCTCAGGGCTTTGG -3’
	R 5’- TGGCAATGGTGTCTTTTCTTTG -3’
IL17A _P2 (-19 to -32) p-STAT3-ChIP	F 5’- CACCTCACACGAGGCACAAG-3’
	R 5’- ATGTTTGCGCGTCCTGATC-3’
IL-17A_P3 (-2913 to -2925) RORγt or HIF1α- ChIP	F 5’- GGCTACAACATAGTCATACAC-3’
	R 5’- GAAGGTATCAGATCCCATTAC-3’
IL-17A_P4 (-6128 to -6140) RORγt or HIF1α- ChIP	F 5’- CAGATGCATGCAGAACTGAC-3’
	R 5’- AGGCTCTGGAGAGCAGACA-3’
IL-17A_P5 (non- RORγt/P-STAT3) binding) RORγt or p-STAT3- ChIP	F 5’- AGTCTGGCCCCTACACACAC-3’
	R 5’- ATGGGGGACTTTTGGGATAG-3’

Table S3. Mouse HCC scoring.

	Features				
Score	Centrilobular vacuolar degeneration	Foci of proliferation	Mitotic rate	Scirrhous type foci of proliferation	Inflammatory cell infiltration
1	<10% of lobules	Small with vacuolated cells (<1 focus / 10X HPF[1])	<1 / 40X HPF[1]	1 or 2 / 10X HPF[1]	<1 / 20x HPF[1]
2	10~33% of lobules	Moderate-sized, multifocal, with small non-vacuolated cells (1 to 2 foci / 10X HPF[1])	1 to 3 / 40X HPF[1]	3 to 6 / 10X HPF[1]	1 to 2 / 20x HPF[1]
3	33~66% of lobules	Moderate-sized and coalescing or large, multifocal (2 to 3 foci / 10X HPF[1])	4 to 6 / 40X HPF[1]	7 to 10 / 10X HPF[1]	2 to 3 /20x HPF[1]
4	>66% of lobules	large and coalescing) >3 foci / 10X HPF[1]	>6 / 40X HPF[1]	>10 / 10X HPF[1]	>3 / 20x HPF[1]

[1] HPF: high-power field.

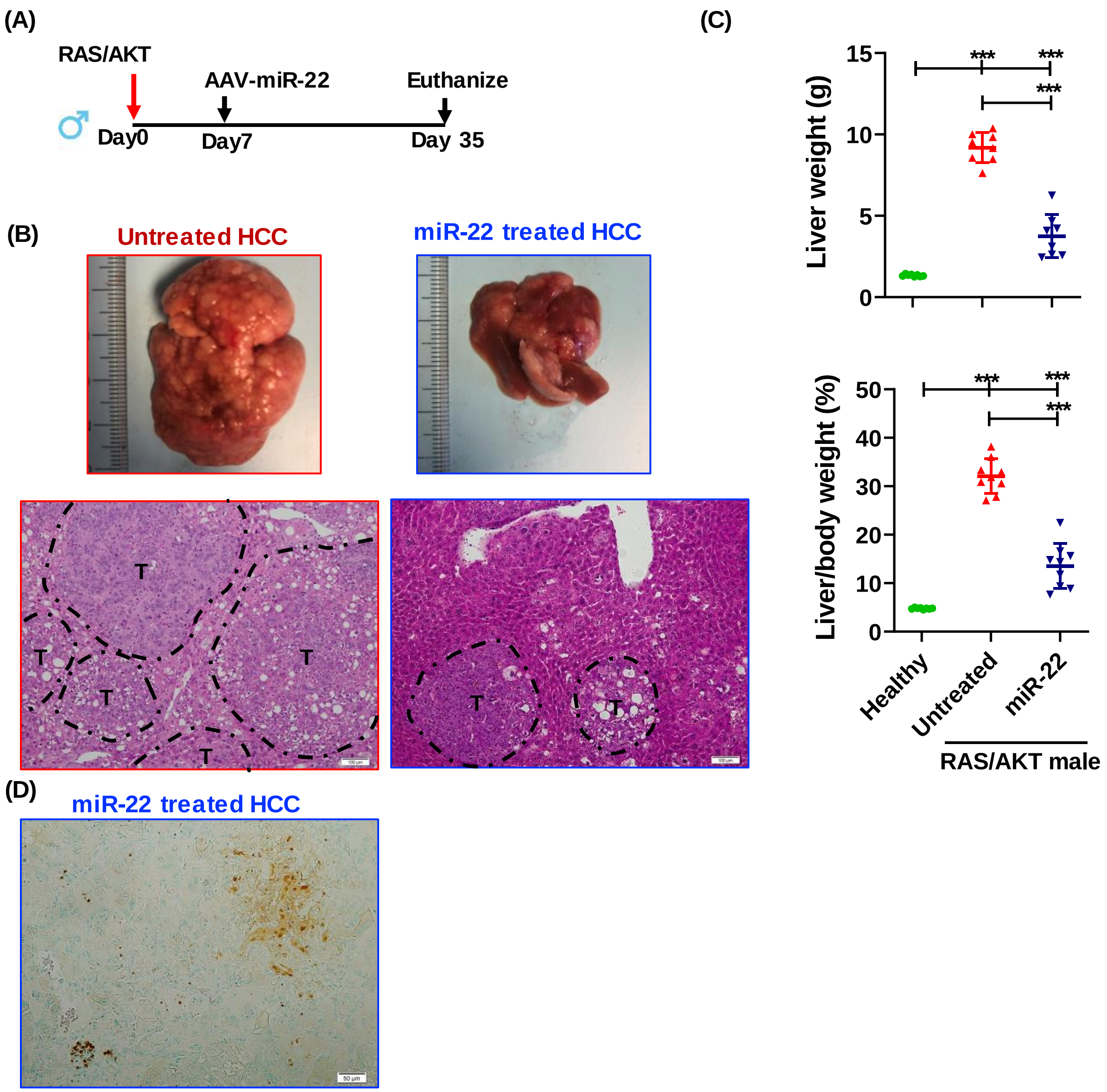

Figure S1. miR-22 treats HCC in male mice. **(A)** Study design for miR-22 treatment in RAS/AKT-induced HCC model, **(B)** Representative liver morphology and H&E-stained liver sections (10X), **(C)** Liver weight and L/B ratio for the studied groups. **(D)** Apoptosis detected by TUNEL staining was found in miR-22-treated HCC female mice as one example (20X).

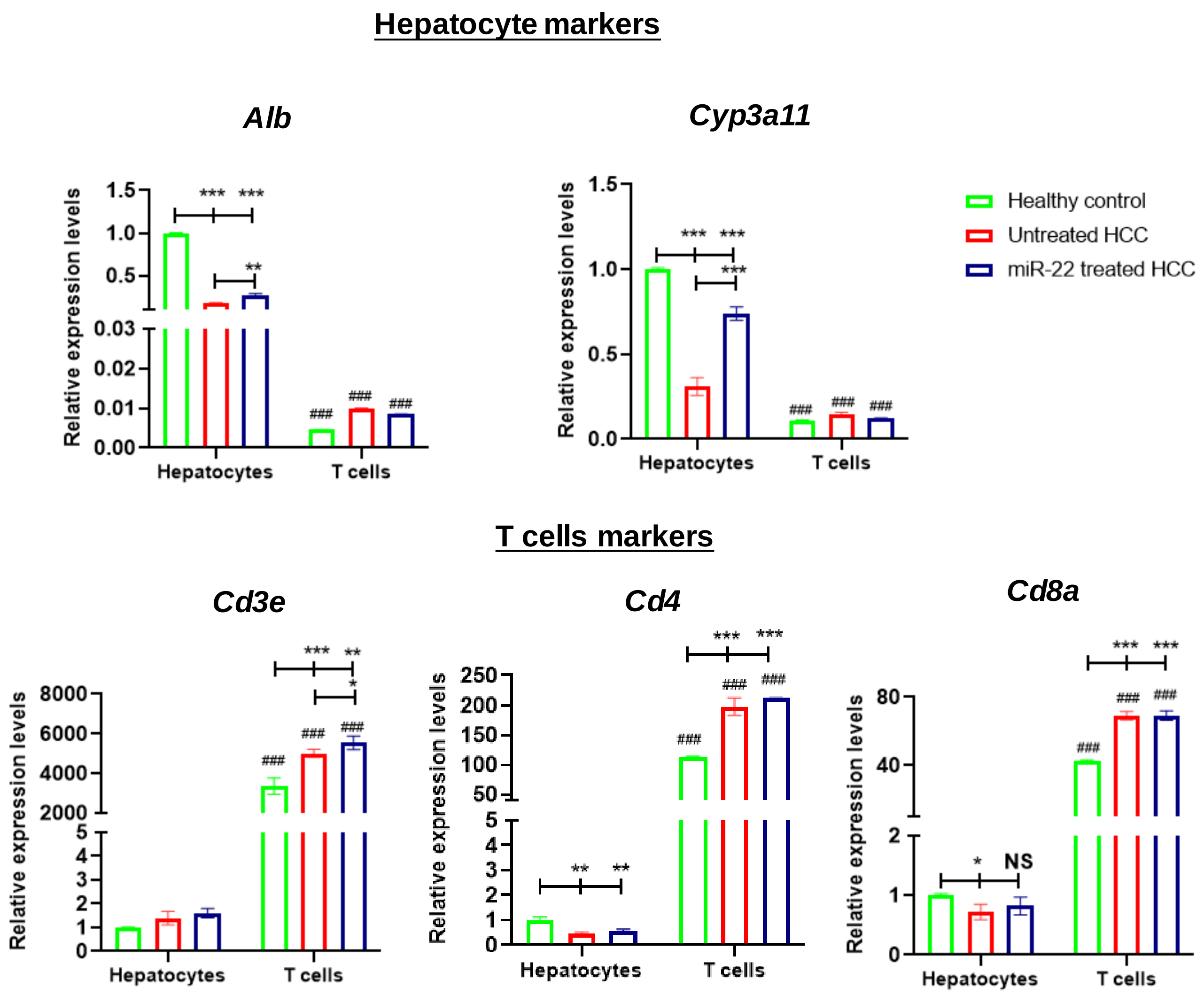

Figure S2. The expression levels of hepatocyte and T cell markers in isolated hepatocytes and T cells. Hepatocytes and T cells isolated from livers of healthy, untreated HCC, and miR-22-treated HCC mice were subjected to RNA extraction followed by qRT-PCR. Data = mean ± SD (n=3). * $p<0.05$, ** $p<0.01$, *** $p<0.001$ by One-way ANOVA. # Comparison between hepatocytes and T cells by unpaired two-tailed Student's t test.

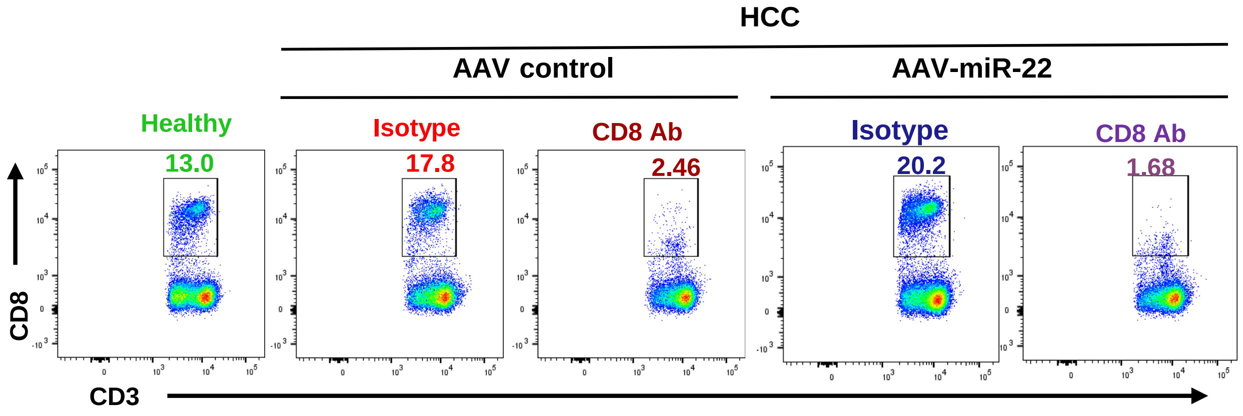

Figure S3. CD8 antibody blockade depletes hepatic CD8⁺ T cells in HCC mice. Representative flow cytometry plots of CD3⁺/CD8⁺ T cells in the livers of healthy, AAV8 control and AAV8-miR-22-treated HCC mice followed by either anti-CD8 mAb or isotype control (n = 4).

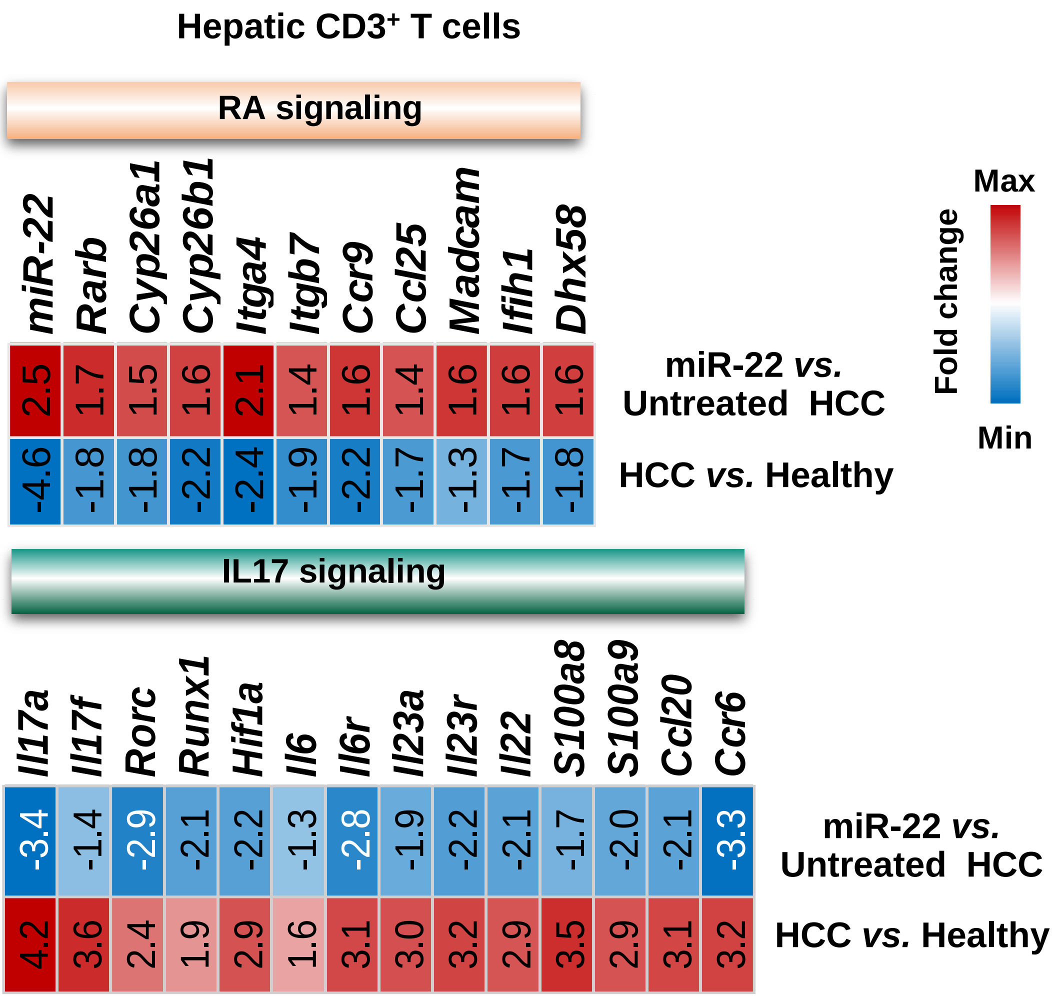

Figure S4. miR-22 increases RA signaling and inhibits IL17 signaling in β-catenin/AKT-driven HCC mice. (A) The fold changes of RA and IL17 signaling-related genes in hepatic T cells were quantified by qRT-PCR and shown in the heatmap. Hepatic T cells isolated from three mice for each group were subjected to RNA extraction followed by qRT-PCR.

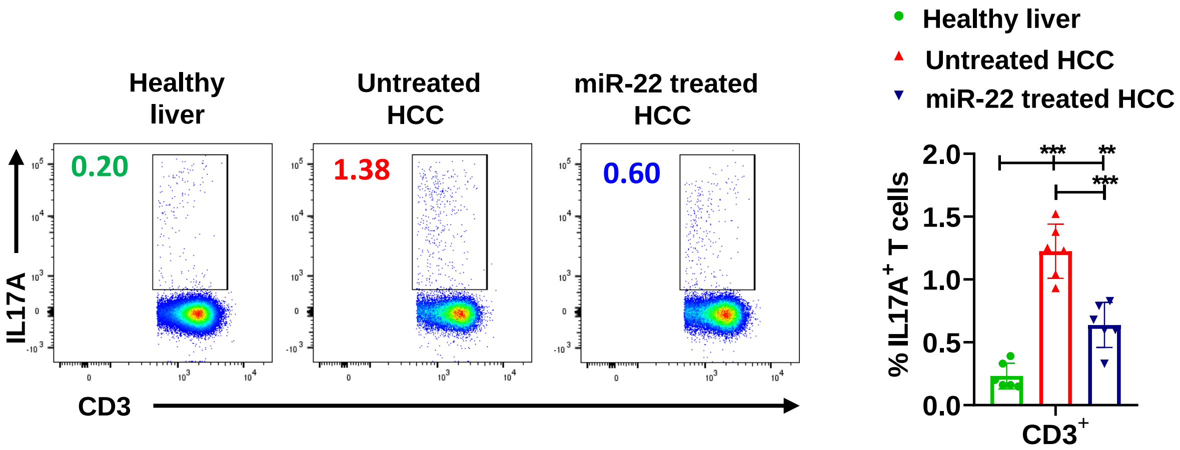

Figure S5. miR-22 treatment reduces IL17-producing T cells. Representative flow cytometry plots and percentage of IL17-producing T cells ($CD3^+IL17A^+$) in the livers of healthy, HCC, and miR-22-treated HCC mice. Data = mean ± SD. ** p<0.01, *** p<0.001 (n = 6 for each group).

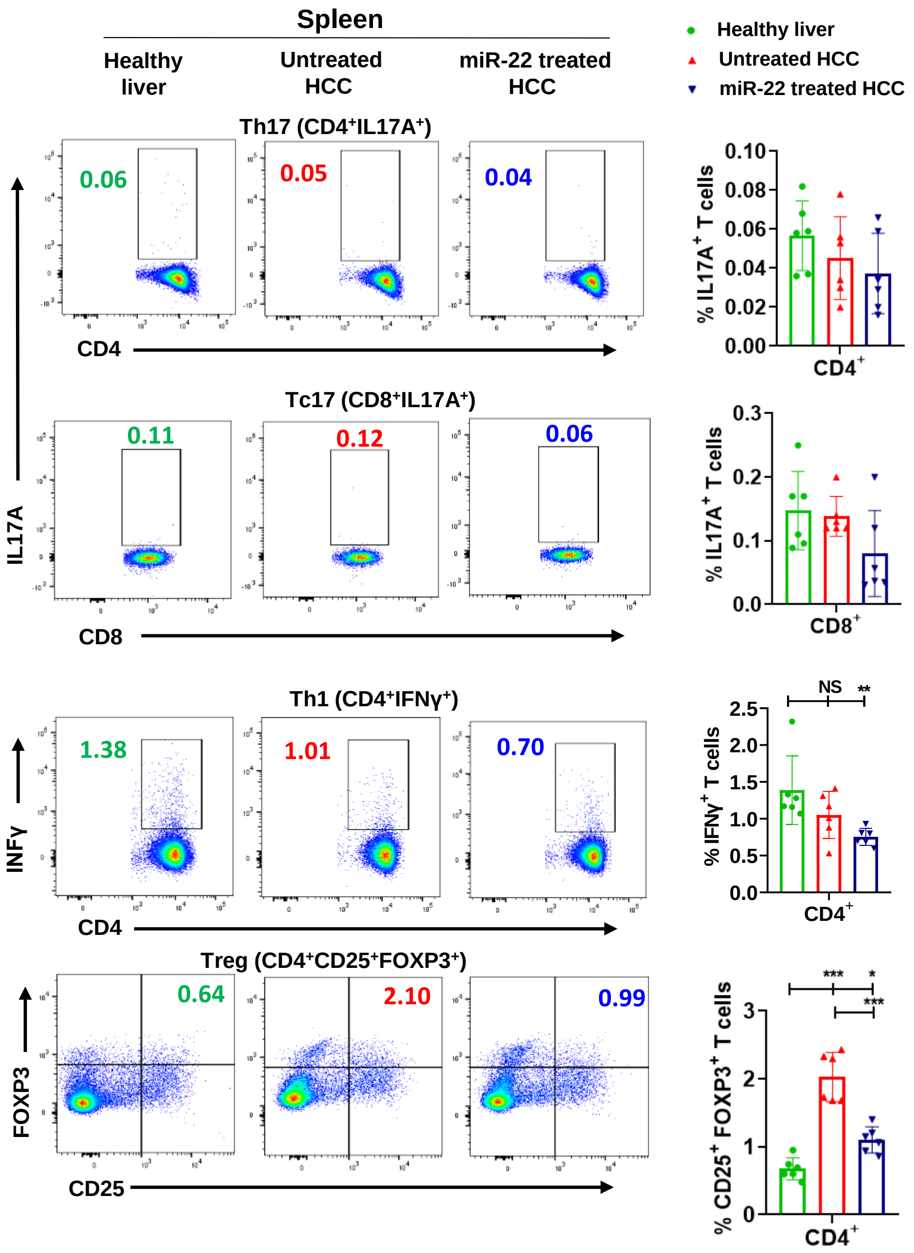

Figure S6. miR-22 treatment reduced Treg and Th1 cells but had no effect on Th17 and Tc17 cells in the HCC splenocytes. Representative flow cytometry plots and percentages of Th17 ($CD4^+IL17A^+$), Tc17 ($CD8^+IL17A^+$), Th1 ($CD4^+IFN\gamma^+$), and Treg ($CD4^+CD25^+FOXP3^+$) T cells from the splenocytes isolated from healthy, untreated HCC, and miR-22-treated HCC mice. Data = mean ± SD. * $p<0.05$, ** $p<0.01$, *** $p<0.001$ by One-way ANOVA (n = 6).

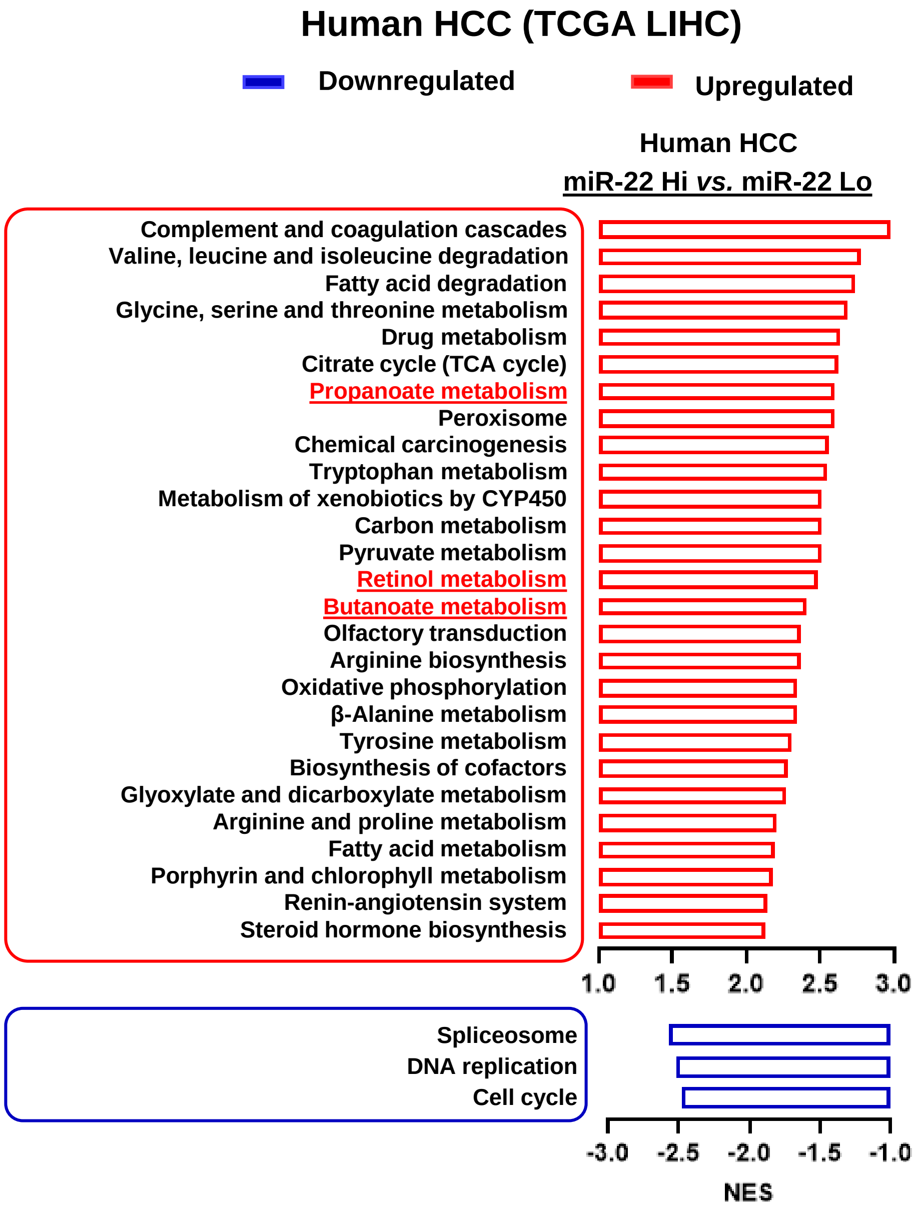

Figure S7. Pathway analysis for the TCGA LIHC human transcriptome profiling based on miR-22 expression levels. Pathways enriched in human HCC by comparing miR-22 high (miR-22 Hi, n=89) *vs*. miR-22 low (miR-22 Lo, n=92) HCC revealed by GSEA based on KEGG gene set. miR-22 inducer signaling (Retinol, Propanoate, and Butanoate metabolism) was underlined and highlighted in red (Upregulated). Normalized Enrichment Score (NES).
